# Targeting a disintegrin and metalloprotease (ADAM) 17-CD122 axis enhances CD8^+^ T cell effector differentiation and anti-tumor immunity

**DOI:** 10.1038/s41392-024-01873-6

**Published:** 2024-06-26

**Authors:** Lina Sun, Anjun Jiao, Haiyan Liu, Renyi Ding, Ning Yuan, Biao Yang, Cangang Zhang, Xiaoxuan Jia, Gang Wang, Yanhong Su, Dan Zhang, Lin Shi, Chenming Sun, Aijun Zhang, Lianjun Zhang, Baojun Zhang

**Affiliations:** 1 Department of Pathogenic Microbiology and Immunology, School of Basic Medical Sciences, Xi’an Jiaotong University, Xi’an, Shaanxi 710061 China; 2 Institute of Infection and Immunity, Translational Medicine Institute, Xi’an Jiaotong University Health Science Center, Xi’an, Shaanxi 710061 China; 3 Key Laboratory of Environment and Genes Related to Diseases, Ministry of Education, Xi’an, Shaanxi 710061 China; 4 Xi’an Key Laboratory of Immune Related Diseases, Xi’an, Shannxi 710061 China; 5 Jiangsu Center for the Collaboration and Innovation of Cancer Biotherapy, Cancer Institute, Xuzhou Medical University, Xuzhou, Jiangsu China; 6 Department of Pediatrics, Qilu Hospital of Shandong University, Jinan, China; 7 National Key Laboratory of Immunity and Inflammation, Suzhou Institute of Systems Medicine, Chinese Academy of Medical Sciences & Peking Union Medical College, Suzhou, 215123 Jiangsu China; 8 Key Laboratory of Synthetic Biology Regulatory Elements, Suzhou Institute of Systems Medicine, Chinese Academy of Medical Sciences & Peking Union Medical College, Suzhou, 215123 Jiangsu China

**Keywords:** Tumour immunology, Lymphocytes

## Abstract

CD8^+^ T cell immune responses are regulated by multi-layer networks, while the post-translational regulation remains largely unknown. Transmembrane ectodomain shedding is an important post-translational process orchestrating receptor expression and signal transduction through proteolytic cleavage of membrane proteins. Here, by targeting the sheddase A Disintegrin and Metalloprotease (ADAM)17, we defined a post-translational regulatory mechanism mediated by the ectodomain shedding in CD8^+^ T cells. Transcriptomic and proteomic analysis revealed the involvement of post-translational regulation in CD8^+^ T cells. T cell-specific deletion of ADAM17 led to a dramatic increase in effector CD8^+^ T cell differentiation and enhanced cytolytic effects to eliminate pathogens and tumors. Mechanistically, ADAM17 regulated CD8^+^ T cells through cleavage of membrane CD122. ADAM17 inhibition led to elevated CD122 expression and enhanced response to IL-2 and IL-15 stimulation in both mouse and human CD8^+^ T cells. Intriguingly, inhibition of ADAM17 in CD8^+^ T cells improved the efficacy of chimeric antigen receptor (CAR) T cells in solid tumors. Our findings reveal a critical post-translational regulation in CD8^+^ T cells, providing a potential therapeutic strategy of targeting ADAM17 for effective anti-tumor immunity.

## Introduction

Upon antigen recognition, CD8^+^ T cells robustly expand to give rise to effector cells to carry out effector functions and memory cells afterward for long-term protection. It is well-appreciated that, depending on different immunological context, CD8^+^ T cells can differentiate into heterogeneous populations.^1^ In response to an acute infection, effector CD8^+^ T cells at the peak of expansion (~day 7) can be divided, based on the expression of KLRG1 and CD127, into two subpopulations: KLRG1^+^CD127^-^ terminally differentiated short-lived effector cells (SLECs) and KLRG1^-^CD127^+^ long-lived memory precursor effector cells (MPECs).^2^ Alternatively, during chronic infection and tumor, CD8^+^ T cells differentiate into exhausted T (Tex) cell trajectory, in which exhausted T (Tex) cells gradually lose cytotoxic function but upregulate inhibitory receptors.^3,4^ Nevertheless, accumulating evidence has revealed that the CD8^+^ stem-like progenitor Tex (Tpex) cells sustain the Tex cell pool^5–8^ and mediate the anti-tumor immunity.^9^

The differentiation and functionality of CD8^+^ T cells are exquisitely regulated by complex networks such as transcription factors (TFs), cytokines, and epigenetic modification.^10^ Orchestrated by the signal cascades of T cell receptor (TCR), co-stimulation, and cytokines, a variety of key TFs are involved in controlling effector, memory, and exhausted differentiation of CD8^+^ T cells, such as T-bet, Eomes, TCF-1, TOX, and BATF.^11–15^ Cytokine-driven signals, particularly, IL-2 and IL-15, which share two receptor subunits CD122 (β chain) and CD132 (γ chain), are crucial for CD8^+^ T cell survival and functionality.^16^ Epigenetic regulations, such as DNA methylation and histone acetylation, modulate the transcription of lineage-specific TFs or key genes of CD8^+^ T cells.^12,17–19^ Post-transcriptional regulations, such as mRNA stability and splicing, and noncoding RNAs, also govern CD8^+^ T cell effector functions.^20–22^ However, regulations at translational/post-translational levels are much less understood in CD8^+^ T cells. The transmembrane ectodomain shedding is a post-translational process of proteolytic cleavage of membrane protein ectodomains, which plays an important role in regulating membrane receptor expression and signal transduction.^23,24^ However, its function in CD8^+^ T cells has not been widely investigated.

The contributing protease for ectodomain shedding is referred to as a sheddase; the A Disintegrin and Metalloprotease (ADAM) family member, ADAM17, is the first characterized sheddase involved in the cleavage of many transmembrane proteins.^25^ To date, more than 90 substrates have been identified for ADAM17, including cytokines (e.g., TNF-α/β and CSF-1), growth factors (e.g., TGFα and EGF), signaling receptors (e.g., IL-6R, Notch1, and TNFR), adhesion molecules (e.g., CD62L and CD44) and enzymes (e.g., ACE-2 and NPR1).^25,26^ Given its broad range of substrates, ADAM17 participates in a wide range of physiological or pathological processes, predominantly in mediating a pro-inflammatory response and promoting tumor development.^25,26^ In addition, ADAM17 plays critical immunological functions in modulating the development, activation, migration, and functionality of various types of immune cells.^26,27^ Specifically in T cells, the expression of ADAM17 on the cell surface and its enzymatic activity are upregulated upon TCR stimulation,^28,29^ which facilitates T cell activation and proliferation by cleavage of Lag-3,^30^ FasL,^31^ CD62L,^32^ CD27^33^ or CD137 (4-1BB).^34^ In autoimmune diseases, ADAM17 promotes T cell overactivation through proteolytical cleavage of a negative immune regulator Pik3ip1.^35^ ADAM17 might negatively correlate with autoimmune disease by inhibiting Th17 cell differentiation through shedding IL-23R.^36,37^

Post-translational regulation in T cells, including ADAM-mediated membrane protein shedding, is easy to ignore, especially with the increasing development of RNA sequencing technology.^27^ Therefore, here, we aim to explore the regulatory mechanisms underlying the differentiation and function of CD8^+^ T cells at the post-translational level by targeting ADAM17. We found that CD8^+^ T cell responses were extensively regulated by post-translational network. T cell-specific deletion of ADAM17 led to a dramatic increase in effector CD8^+^ T cell program and cell survival. ADAM17 ablation enhanced the anti-tumor activity of CD8^+^ T cells. Mechanistically, ADAM17 cleaves surface CD122 on CD8^+^ T cells. Inhibition of ADAM17 improved the anti-tumor effects of chimeric antigen receptor (CAR) T cells in solid tumor. Finally, the regulatory mechanism of CD122 by ADAM17 was verified in human CD8^+^ T cells, highlighting the potential application in cancer immunotherapy.

## Results

### Post-transcriptional/translational regulation in CD8^+^ T cell immune response

**Fig. 1:**
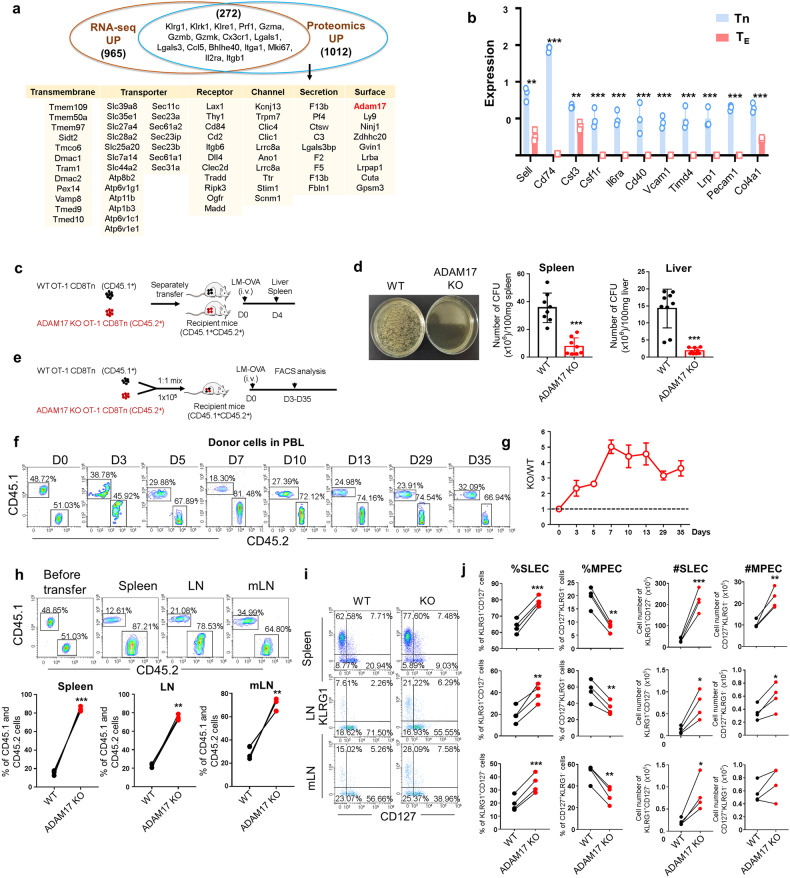
ADAM17 deficiency enhances effector CD8^+^ T cell response. **a** Venn diagram depicting the overlapping portion of upregulated genes between naïve CD8^+^ T cells and KLRG1^+^CD127^-^ CD8^+^ SLECs based on RNA-Seq (red circle) and proteomic analysis (blue circle). Representative overlapping genes were shown and representative membrane proteins belonging to different categories were listed. **b** The protein levels of known ADAM17 substrates in naïve (Tn) and effector (T_E_) CD8^+^ T cells were shown based on the proteomic results. **c** Experimental schematic of in vivo CD8^+^ T cell adoptive transfer assay to determine the bacterial clearance. Bacterial loads were determined 4 days after infection in the spleen and liver (*n* = 8). **d** Representative photos of agar plates showing the bacterial colony formation in the spleen; numbers of bacterial colonies per 100 mg spleen or liver from mice received WT or ADAM17 KO CD8^+^ T cells. **e** Experimental schematic of CD8^+^ T cell co-transfer assay. Donor cells were detected by flow cytometry analysis in the recipient mice at indicated timepoints post-infection (*n* = 4). (**f**) Representative FACS plots of WT (CD45.1^+^) and KO (CD45.2^+^) cells in CD8^+^ T cells in peripheral blood lymphocytes (PBL) at different timepoint after *LM*-OVA infection. **g** The ratio of donor cell frequency of KO to WT cells in the PBL at different time points. **h** Representative FACS plots and the frequencies of WT (CD45.1^+^) and KO (CD45.2^+^) cells in CD8^+^ T cells before transfer and 7 days after *LM*-OVA infection in spleen, peripheral lymph nodes (LNs) and mesenteric LNs (mLNs). **i** Representative FACS plots of KLRG1 and CD127 expression in splenic CD8^+^ T cells in spleen, LNs and mLNs 7 days post-infection. **j** The frequency (%) and cell number (#) of KLRG1^+^CD127^-^ (SLEC) and KLRG1^-^CD127^+^ (MPEC) cells in CD8^+^ T cells in spleen, LNs and mLNs 7 days post-infection. Data are shown as the mean ± SD. Statistical testing is depicted as two-sided, unpaired t-tests; *P ≤ 0.05, **P ≤ 0.01, ***P ≤ 0.001

Transcriptomic analysis, as one of the important systems biology methods, has been extensively used to characterize T cell phenotypes and functionality at the RNA level.^10^ To explore the effects of post-transcriptional and post-translational regulation on CD8^+^ T cells, we performed parallel transcriptomic and proteomic analysis of naïve and KLRG1^+^ effector CD8^+^ T cells obtained from an in vivo acute infection model. Naïve (CD62L^+^CD44^-^) CD8^+^ T cells were isolated from OT-1 transgenic mice in which the T cells express the ovalbumin (OVA) peptide SIINFEKL-specific TCR and adoptively transferred into the wild-type (WT) recipient mice followed by infection with *Listeria monocytogenes* (*LM*) expressing OVA (*LM*-OVA) for 7 days. Naïve CD8^+^ T cells and KLRG1^+^CD127^-^ CD8^+^ SLECs were sorted by flow cytometry (supplementary Fig. 1a) and subjected to RNA sequencing (RNA-seq) and mass spectrometry (MS)-based proteomics, respectively. A total number of 6331 and 6126 proteins were identified in naïve CD8^+^ T cells and CD8^+^ SLECs, respectively. Compared to naïve CD8^+^ T cells, there were 1284 upregulated proteins (greater than 1.5-fold, *P* < 0.05) in KLRG1^+^CD127^-^ CD8^+^ SLECs (Fig. 1a and Supplementary Data 1). Comparing the transcriptomes, we found that 1237 genes were upregulated (log_2_ fold-change greater than 1, *P* < 0.05) in CD8^+^ SLECs (Fig. 1a and Supplementary Data 2). When comparing the differentially expressed genes (DEGs) obtained from RNA-seq data and the differentially expressed proteins (DEPs) from proteomics analysis, we surprisingly found that only a small proportion of genes (272) were overlapped, including well-known signature effector molecules of CD8^+^ T cells (*Klrg1*, *Prf1*, *Gzma*, *Gzmb*, *Cx3cr1*, *Lgals1*, *Ccl5*, *Mki67*, *Il2ra* etc.) (Fig. 1a and Supplementary Data 3). Notably, those non-overlapped proteins contain a great number of cell membrane proteins, such as transmembrane proteins, signaling receptors, transporters, ion channels, secreted molecules and other surface proteins (Fig. 1a and Supplementary Data 3), suggesting that post-transcriptional/translational regulations highly participate in CD8^+^ T cell immune response.

### ADAM17 deficiency enhances effector CD8^+^ T cell differentiation

The proteolytic cleavage of transmembrane proteins (a process referred to as “ectodomain shedding”) is a critical post-transcriptional/translational modification. Interestingly, ADAM17, one of the ADAM family of metalloproteinases, which are key executors for ectodomain shedding,^38^ was also identified in the upregulated proteins from MS result (Fig. 1a). Meanwhile, many known ADAM17 substrates^25,26^ were decreased at the protein level (Fig. 1b). Therefore, we next aim to investigate the immunological function of ADAM17 in effector CD8^+^ T cell differentiation and function during immune response. To this end, T cell-specific *ADAM17* knockout (KO) mice (ADAM17^fl/fl^Lck-Cre^*+*^(OT-1^+^)) were developed by crossing *ADAM17* flox mice with Lck-Cre transgenic strain, and were further crossed with OT-1 transgenic mice (supplementary Fig. 1b and c). The qPCR and Western blot results confirmed the complete deletion of ADAM17 at both mRNA and protein levels in CD8^+^ T cells from KO mice compared to WT (ADAM17^fl/fl^Lck-Cre^-^) mice (supplementary Fig. 1d and e). Consistent with a previous study,^39^ T cell deficient in ADAM17 did not affect T cell development and function under homeostatic status (supplementary Fig. 2).

Given the importance of effector CD8^+^ T cells in protecting against pathogenic infection, we first examined ADAM17 deficiency in CD8^+^ T cells in an acute infection model. As illustrated in Fig. 1c, naïve CD8^+^ T cells from WT (OT-1^+^CD45.1^+^) and ADAM17 KO (OT-1^+^CD45.2^+^) mice were isolated and separately transferred into the CD45.1^+^CD45.2^+^ WT recipient mice by intravenous injection. Following infection with *LM*-OVA, bacterial load was determined in the spleen and liver. Surprisingly, compared to mice transferred with WT CD8^+^ T cells, those with ADAM17 KO CD8^+^ T cells had significantly reduced bacterial colonies on agar plates in both spleen and liver 4 days (Figs. 1d) and 7 days after infection (supplementary Fig. 3a), suggesting an enhanced effector function of ADAM17 deficient CD8^+^ T cells. To determine the intrinsic role of ADAM17 in CD8^+^ T cells during the immune response, we further co-transferred naïve OT-1^+^ CD8^+^ T cells from WT (CD45.1^+^) and ADAM17 KO (CD45.2^+^) mice at a ratio of 1:1 into the same CD45.1^+^CD45.2^+^ WT recipient mice. The antigen-specific CD8^+^ T cell response and maintenance were assessed in the secondary lymphoid tissue following *LM*-OVA infection (Fig. 1e). The results showed that the ratio of ADAM17 KO to WT CD8^+^ T cells was remarkably increased in the peripheral blood lymphocytes (PBL) after infection compared to an equal number transferred (Fig. 1f and g). Consistently, ADAM17 deficient CD8^+^ T cells exhibited a significant increase in cell expansion during the acute infection on day 7 in the spleen, peripheral lymph nodes (LNs) and mesenteric LN (mLNs) compared to WT counterparts (Fig. 1h), suggesting a greatly augmented generation of antigen-specific effector CD8^+^ T cells. Further examination of the CD8^+^ T cell differentiation revealed that the frequency of KLRG1^+^CD127^-^ SLECs was significantly increased and the KLRG1^-^CD127^+^ MPECs were decreased, even though their absolute cell number was all increased due to the massively elevated KO donor cells (Fig. 1i and j). These data elucidate that ADAM17 deficiency enhances effector CD8^+^ T cell differentiation and functionality.

### ADAM17 deficiency increases cell survival and effector signature of CD8^+^ T cells

**Fig. 2:**
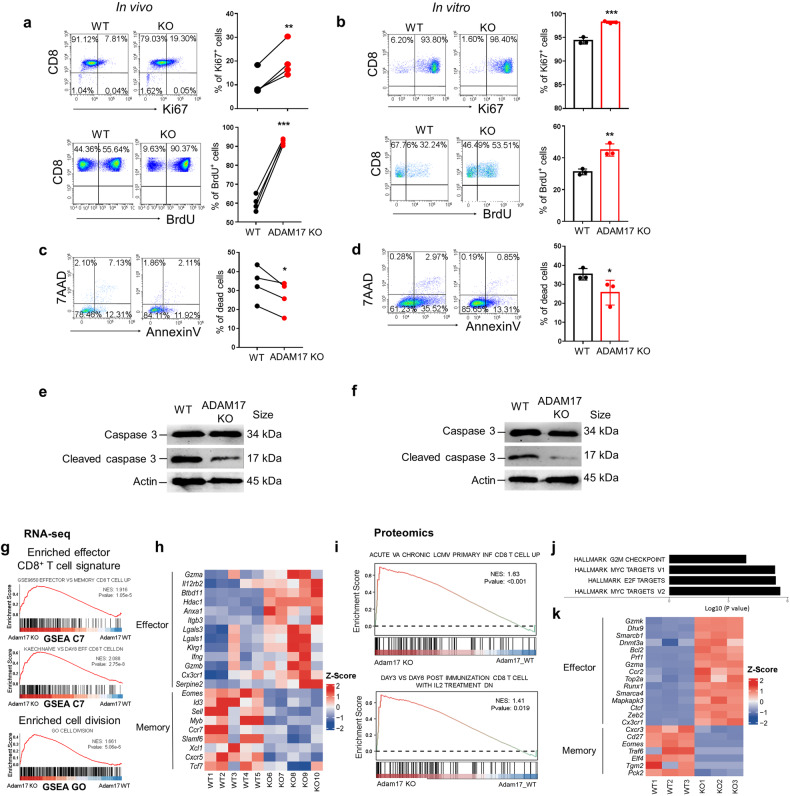
ADAM17 deletion leads to increased cell survival and effector signature of CD8^+^ T cells. **a** Representative FACS plots and the frequencies of Ki67 and BrdU expression in WT and KO donor CD8^+^ T cells in the spleen from recipient mice 7 days post *LM*-OVA infection in the in vivo co-transfer model (*n* = 4). **b** Splenocytes from WT (ADAM17^fl/fl^Lck-Cre^-^) and ADAM17 KO (ADAM17^fl/fl^Lck-Cre^+^) mice were isolated and stimulated in vitro with anti-CD3/CD28 antibodies for 72 hr to measure cell proliferation. Representative FACS plots and the frequencies of Ki67 and BrdU expression in WT and KO CD8^+^ T cells (*n* = 3). **c**, **d** Representative FACS plots and the frequencies of Annexin V and 7AAD expression in WT and ADAM17 KO CD8^+^ T cells from the in vivo co-transfer model (**c**) and in vitro stimulation for 48 hr (**d**). **e**–**f** The protein levels of total caspase 3 and cleaved caspase 3 were measured by Western blot in WT and ADAM17 KO CD8^+^ T cells from the in vivo co-transfer model (**e**) and in vitro stimulation for 48 hr (**f**). **g**–**k** In the co-transfer model, WT and ADAM17 KO CD8^+^ T cells from recipient mice 7 days after *LM*-OVA infection were sorted for RNA-seq analysis (*n* = 5) and proteomic analysis (*n* = 3). **g** Gene set enrichment analysis (GSEA) plots depict the gene sets enriched in ADAM17 KO CD8^+^ T cells by RNA-seq were associated with effector CD8^+^ T cell signature and cell division-associated signaling pathway. **h** Heatmap exhibits the DEGs associated with effector and memory CD8^+^ T cell differentiation between WT and ADAM17 KO groups. **i** GSEA plots depict the protein sets enriched in ADAM17 KO CD8^+^ T cells by proteomics were associated with effector CD8^+^ T cell signature. **j** Hallmark protein sets enriched in ADAM17 KO CD8^+^ T cells were G2M checkpoint, E2F, and MYC targets. **k** Heatmap exhibits the DEPs associated with effector and memory CD8^+^ T cells between WT and ADAM17 KO groups. Heatmap colors represent the z-score values relative to the control (**h** and **k**). Data are representative of three independent experiments shown as the mean ± SD. Statistical testing is depicted as two-sided, unpaired t-tests; *P ≤ 0.05, **P ≤ 0.01, ***P ≤ 0.001

Upon antigen recognition, naïve CD8^+^ T cells undergo a robust expansion followed by massive apoptosis, a process named contraction, before memory T cell development.^1^ We then sought to determine whether the increase of antigen-specific effector CD8^+^ T cells after ADAM17 deletion was caused by increased cell proliferation or decreased cell apoptosis. The cell proliferation and apoptosis in CD8^+^ T cells were assessed in the co-transfer model as in Fig. 1e. ADAM17 KO CD8^+^ T cells, compared to WT controls, showed elevated cell proliferation evidenced by higher expression of cell-cycle marker Ki67 and BrdU on day 7 (Fig. 2a), while the slightly decreased percentage of apoptotic cells as indicated by Annexin V and 7AAD staining (Fig. 2c). Furthermore, when stimulated in vitro with anti-CD3/CD28 antibodies, ADAM17 deficient CD8^+^ T cells also exhibited increased cell proliferation and decreased apoptosis compared to WT cells (Fig. 2b and d). The reduction of cell apoptosis was further verified by the decreased expression of the major apoptotic caspase, cleaved caspase 3 by Western blot in ADAM17 deficient CD8^+^ T cells from both in vivo co-transfer model (Fig. 2e) and in *vitro* stimulation (Fig. 2f). However, the T cell activation was unaffected in ADAM17 KO CD8^+^ T cells, evidenced by comparable expression of activation markers CD69 and CD25 (supplementary Fig. 3b), as well as canonical TCR signaling pathways (e.g. ERK and NF-κB pathways) (supplementary Fig. 3c). These results demonstrate that ADAM17 deletion has no impact on T cell activation but increases cell survival of CD8^+^ T cells after antigen stimulation.

To understand how ADAM17 deficiency affects CD8^+^ T cell properties, comprehensive genome-wide transcriptomic analysis (RNA-seq) was performed to compare the transcriptomic signatures of WT and ADAM17 KO CD8^+^ T cells from recipient mice 7 days after *LM*-OVA infection in the co-transfer model. Gene set enrichment analysis (GSEA) of the DEGs between WT and ADAM17 KO CD8^+^ T cells revealed that the effector signatures of CD8^+^ T cells, as well as cell division-associated signaling pathway were significantly enriched in ADAM17 deficient CD8^+^ T cells (Fig. 2g). Consistently, the expression of prominent genes associated with effector CD8^+^ T cells (*Gzma, IL12rb2, Klrg1, Lgals1, Ifng, Gzmb* and *Cx3cr1*) were highly upregulated while memory CD8^+^ T cell-associated molecules (*Eomes, Id3, Sell, Myb, Ccr7, Slamf6* and *Tcf7*) were downregulated after ADAM17 deficiency (Fig. 2h and Supplementary Data 4). Furthermore, proteomic analysis of total proteins extracted from WT and ADAM17 KO CD8^+^ T cells from the co-transfer model on day 7 showed that proteins associated with effector function of CD8^+^ T cells were highly enriched in ADAM17 KO group (Fig. 2i). GSEA of the hallmark pathways identified a significantly enrichment of proteins involved in cell division and growth-related pathways, such as G2M, E2F and MYC targets in ADAM17 KO CD8^+^ T cells (Fig. 2j). Similarly, genes associated with effector CD8^+^ T cell signature (*Gzmk, Gzma, Prf1, Ccr2, Zeb2,* and *Cx3cr1*) were also highly upregulated while memory CD8^+^ T cell molecules (*Cxcr3, Cd27, Traf6, Elf4* and *Eomes*) were downregulated at the protein level after ADAM17 deletion (Fig. 2k and Supplementary Data 5). Notably, some recently identified regulators for effector function of CD8^+^ T cells were also increased in ADAM17 deficient CD8^+^ T cells, such as DNA/RNA helicase Dhx9,^40^ DNA methyltransferase Dnmt3a,^41^ cBAF components Smarcb1 and Smarca4,^42^ and chromatin remodeler CTCF.^43^ Moreover, effector CD8^+^ T cell markers KLRG1, CX3CR1, ICOS, and T-bet were also upregulated in ADAM17 KO KLRG1^+^CD127^-^ SLECs by flow cytometry (supplementary Fig. 3d). Together, these data indicate that ADAM17 plays a critical role in regulating effector CD8^+^ T cell program.

### ADAM17 deficiency enhances the anti-tumor activity of CD8^+^ T cells

**Fig. 3:**
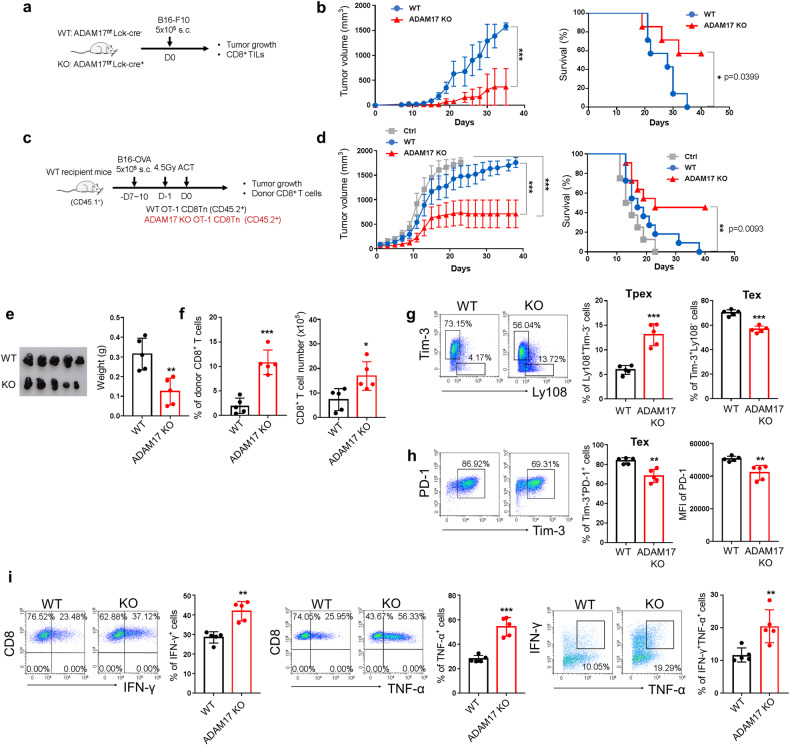
ADAM17 deletion enhances the anti-tumor activity of CD8^+^ T cells. Schematic diagram showing the experimental design of the direct tumor model (**a**) and the adoptive cell transfer (ACT) tumor model (**c**). Tumor growth and survival of tumor-bearing mice were monitored over time in the direct tumor model (**b**) (*n* = 7) and ACT tumor model (**d**) *(n* = 10). **e** Representative photo (left) and the weight (right) of tumor tissues obtained from mice that received either WT or ADAM17 KO OT-1^+^ CD8^+^ T cells (*n* = 5). **f** Percentage of WT and KO donor CD8^+^ T cells in tumor-infiltrating immune cells and the absolute cell number of donor CD8^+^ T cells in WT and KO groups. **g** Representative FACS plots of Tim-3 and Ly108 expression in WT and KO OT-1^+^ TILs (left). The frequencies of Ly108^+^Tim-3^-^ and Tim-3^+^Ly108^-^ cells in OT-1^+^ TILs (right). **h** Representative FACS plots of PD-1 and Tim-3 expression in WT and KO OT-1^+^ TILs (left). The frequencies of Tim-3^+^PD-1^+^ cells and the mean fluorescence intensity (MFI) of PD-1 in OT-1^+^ TILs (right). **i** Representative FACS plots of WT and KO OT-1^+^ TILs producing cytokines IFN-γ and TNF-α (left). The frequencies of IFN-γ^+^, TNF-α^+^ and IFN-γ^+^TNF-α^+^ CD8^+^ T cells in WT and KO OT-1^+^ TILs (right). Data are shown as the mean ± SD. Statistical testing is depicted as two-sided, unpaired t-tests; *P ≤ 0.05, **P ≤ 0.01, ***P ≤ 0.001

Effector CD8^+^ T cells are a crucial T cell population to eliminate malignant cells in cancer.^44^ Thus, we next evaluated the role of ADAM17 in regulating the anti-tumor activity of CD8^+^ T cells. First, melanoma B16-F10 cells were inoculated directly onto ADAM17 WT (ADAM17^fl/fl^Lck-Cre^-^) and KO (ADAM17^fl/fl^Lck-Cre^+^) mice (Fig. 3a). The results showed that mice with T cell-specific knockout ADAM17 had remarkably diminished tumor growth and prolonged mouse survival rate compared to WT control mice (Fig. 3b and supplementary Fig. 4a). Therefore, the tumor weight was reduced in ADAM17 KO mice, accompanied by an increased percentage and cell number of CD8^+^ tumor-infiltrating lymphocytes (TILs) (supplementary Fig. 4b). Generally, CD8^+^ Tex cells in tumor microenvironment comprise heterogeneous populations with a sequential differentiation trajectory of naïve cells, Ly108^+^Tim-3^-^ stem-like CD8^+^ Tpex cells, Tim-3^+^Ly108^-^ intermediate Tex cells and Tim-3^+^PD-1^+^ terminal Tex cells.^10^ Moreover, the frequency of Ly108^+^Tim-3^-^ CD8^+^ Tpex cells was slightly elevated, whereas the Tim-3^+^Ly108^-^ intermediate Tex cells and Tim-3^+^PD-1^+^ terminal Tex cells were reduced, and IFN-γ producing CD8^+^ TILs were increased in ADAM17 KO mice (supplementary Fig. 4c and d), indicating a favorable anti-tumor immunity in T cell-specific ADAM17 KO mice. To verify the anti-tumor ability of ADAM17 KO CD8^+^ T cells, adoptive cell transfer (ACT) experiments were performed. CD8^+^ T cells (OT-1^+^) from WT and ADAM17 KO mice were transferred into mice bearing B16 cells expressing OVA antigen (B16-OVA) (Fig. 3c). In agreement with the previous results in the direct tumor model, ADAM17 ablation in CD8^+^ T cells exhibited a robust anti-tumor activity (Fig. 3d and supplementary Fig. 4e). Transfer of ADAM17 deficient CD8^+^ T cells significantly slowed the tumor growth and improved the survival rate in mice compared to mice that received no T cells or WT T cells (Fig. 3d and supplementary Fig. 4e). Similarly, the tumor size and weight were decreased while the percentage and cell number of OT-1^+^ CD8^+^ TILs were increased in mice that received ADAM17 KO CD8^+^ T cells (Fig. 3e and f). Analysis of OT-1^+^ CD8^+^ TILs revealed that ADAM17 ablation in CD8^+^ T cells displayed remarkably elevated frequency of Ly108^+^Tim-3^-^ Tpex cells and decreased Tim-3^+^Ly108^-^ intermediate Tex cells and Tim-3^+^PD-1^+^ terminal Tex cells, as well as downregulated expression level of PD-1 (Fig. 3g and h). In addition, ADAM17 deficient CD8^+^ TILs exhibited enhanced production of both cytokines IFN-γ and TNF-α compared to their WT counterparts (Fig. 3i). Moreover, the IFN-γ^+^TNF-α^+^ polyfunctional effector CD8^+^ T cells were also increased after ADAM17 deficiency (Fig. 3i). Collectively, ADAM17 deficiency favors the anti-tumor activity of CD8^+^ T cells.

### Upregulation of CD122 is associated with enhanced CD8^+^ T cell function after ADAM17 deficiency

As an essential member of ectodomain sheddase, ADAM17 executes its biological functions mainly through the cleavage of membrane substrates.^45^ In line with this notion, Gene Ontology (GO) analysis of RNA-seq data indicated that in ADAM17 deficient CD8^+^ T cells, genes associated with adhesion pathways, such as cell-substrate, cell-matrix, and cell-cell adhesion, were significantly enriched (supplementary Fig. 5a). Additionally, shedding of CD62L and TNF-α which are well-known ADAM17 substrates^28^ was impaired in ADAM17 depleted CD8^+^ T cells as their expression was retained on the cell surface after T cell activation stimulated in vitro (supplementary Fig. 5b), which also demonstrated the successful ADAM17 depletion in KO cells. Therefore, to identify the target genes of ADAM17 in regulating effector CD8^+^ T cells, we performed proteomic analysis of enriched membrane proteins extracted from WT and ADAM17 KO CD8^+^ T cells isolated from recipient mice 7 days after *LM*-OVA infection in the co-transfer model. Among the 5536 proteins identified, 922 proteins were upregulated, and 911 proteins were downregulated (greater than 1.5-fold, P < 0.05) in ADAM17 KO cells (supplementary Fig. 5c and Supplementary data 6). GO analysis of the DEPs revealed that the enriched biological functions in ADAM17 KO CD8^+^ T cells were associated with membrane-bound vacuole and organelles, vesicle-associated transport, GTPase activity, and membrane invagination, whereas downregulated pathways were ribosome activity and RNA process (supplementary Fig. 5d and e).

**Fig. 4:**
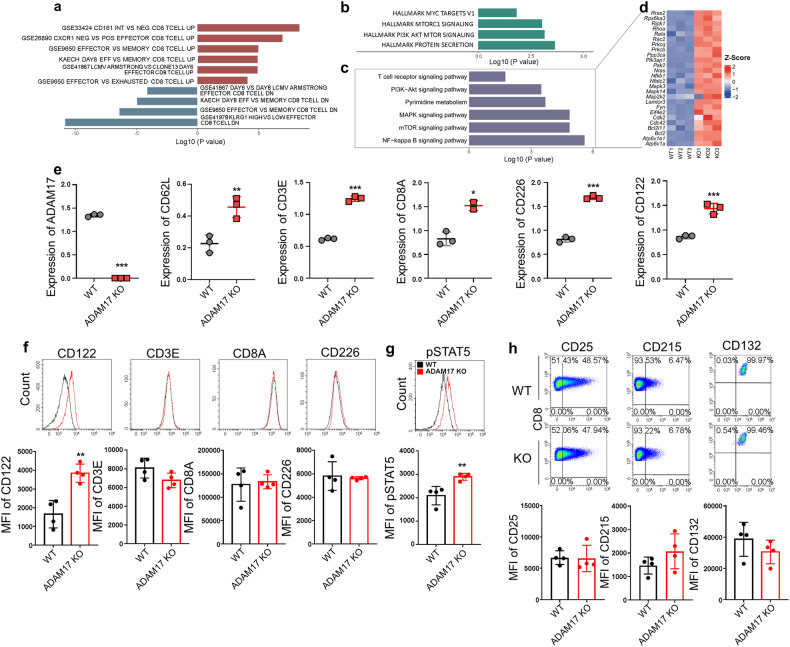
Upregulated CD122 is associated with enhanced CD8^+^ T cell function after ADAM17 deficiency. **a** In the co-transfer model, WT and ADAM17 KO CD8^+^ T cells from recipient mice 7 days after *LM*-OVA infection were sorted and extracted membrane proteins for proteomic analysis (*n* = 3). GSEA plots depict the C7 immunologic gene sets enriched in ADAM17 KO CD8^+^ T cells. **b** Hallmark gene sets enriched in ADAM17 KO CD8^+^ T cells were MYC targets, PI3K-Akt-mTOR signalings. **c** KEGG gene sets enriched in ADAM17 KO CD8^+^ T cells were indicated, and (**d**) heatmap displays representative DEPs associated with these KEGG pathways. **e** The protein levels of ADAM17, CD62L, CD3E, CD8A, CD226, and CD122 in WT and ADAM17 KO CD8^+^ T cells based on proteomics data. **f** Representative FACS histograms of CD122, CD3E, CD8A, and CD226 expression in WT and ADAM17 KO CD8^+^ OT-1^+^ TILs (up). The MFI levels of CD122, CD3E, CD8A and CD226 in WT and ADAM17 KO OT-1^+^ TILs (bottom) (*n* = 4). **g** WT and ADAM17 KO OT-1^+^ TILs were isolated and stimulated with IL-2 for 10 min. Representative FACS plots (up) and MFI of pSTAT5 in WT and ADAM17 KO OT-1^+^ TILs (bottom). **h** Representative FACS plots (up) and MFI of CD25, CD215 and CD132 expression in WT and ADAM17 KO OT-1^+^ TILs (bottom). Data are representative of three independent experiments shown as the mean ± SD. Statistical testing is depicted as two-sided, unpaired t-tests; *P ≤ 0.05, **P ≤ 0.01, ***P ≤ 0.001

Pathway enrichment analysis of DEPs between WT and ADAM17 KO CD8^+^ T cells based on C7 immunologic gene sets and hallmark gene sets demonstrated that effector CD8^+^ T cell program and cell growth-related pathways were markedly enriched in ADAM17 ablated CD8^+^ T cells (Fig. 4a and b). Furthermore, Kyoto Encyclopedia of Genes and Genomes (KEGG) enrichment analysis showed that pathways and featured genes of TCR signaling, PI3K-Akt-mTOR signaling, MAPK signaling and NF-κB signaling were significantly enriched (Fig. 4c and d), indicating an enhancement of effector T cell signature in ADAM17 deficient CD8^+^ T cells. To gain more insight into the direct targets of ADAM17, we focused on the membrane molecules associated with positive regulations of effector T cell function, such as TCR signaling and costimulatory molecules. Of note, most of known TCR and costimulatory receptors were unchanged, such as TCRα, CD3D, CD3G, ZAP70, Lck, Lat, CD5, CD6, CD28, CD27, CD2, GITR, HEVM, and CD150 (Supplementary data 7). ADAM17 deletion, evidenced by a significant protein reduction (Fig. 4e), led to the increased protein level of CD62L (encoded by *Sell*) in ADAM17 KO CD8^+^ T cells, accompanied with elevated protein expression of a few TCR and costimulatory molecules, such as CD3E, CD8A, CD226 and CD122 (encoded by *Il2rb*) (Fig. 4e). It is noteworthy that these proteins are physically interacted with ADAM17 on the cell surface according to the interactome data from a recent study.^46^ However, most of their expression patterns were inconsistent between mRNA and protein levels based on RNA-seq and proteomics data (supplementary Fig. 6a and Fig. 4e), supporting the evidence of post-transcriptional/translational regulation in CD8^+^ T cells. To further explore their function in mediating the enhanced anti-tumor immunity of ADAM17 deficient CD8^+^ T cells, we examined their expression in WT and ADAM17 deficient CD8^+^ TILs in the ACT tumor model (Fig. 3c). Interestingly, only CD122, but not CD3E, CD8A and CD226, was significantly increased after ADAM17 deletion (Fig. 4f). Similar results were also seen in WT and ADAM17 KO CD8^+^ TILs from the direct tumor model (supplementary Fig. 6b), as well as in KLRG1^+^CD127^-^ CD8^+^ SLECs from the in vivo acute infection model (supplementary Fig. 6c). Consistently, the phosphorylation of STAT5 (pSTAT5), a transcription factor downstream of CD122 signaling, was increased in ADAM17 depleted CD8^+^ TILs after IL-2 stimulation ex vivo (Fig. 4g). Considering that CD122 is the receptor β chain, shared by IL-2 and IL-15, which are crucial cytokines for CD8^+^ T cell proliferation and function,^47,48^ and those cytokine receptors have been shown to be regulated by proteolytic cleavage,^49,50^ we next investigated the expression of other IL-2/15 receptors—IL-2Rα (CD25), IL-15Rα (CD215) and IL-2/15Rγ (CD132)—in CD8^+^ TILs. It showed that the expression of CD25, CD215, and CD132 did not change after ADAM17 deletion (Fig. 4h). Of note, the expression of checkpoint receptors Lag-3 and Tim-3, which are known ADAM17 substrates^30,51,52^ was unchanged or slightly decreased in ADAM17 deficient CD8^+^ T cells (supplementary Fig. 6d and e). Given that ADAM17-mediated substrate cleavage is highly dependent on the biological context,^27^ the regulation of Lag-3 and Tim-3 by ADAM17 in tumor-infiltrating CD8^+^ T cells might have more complex mechanisms. Taken together, ADAM17 ablation in CD8^+^ T cells leads to an increased expression of CD122, which is likely associated with the enhanced effector CD8^+^ T cell response.

### ADAM17 regulates effector CD8^+^ T cell survival and function by cleavage of CD122

**Fig. 5:**
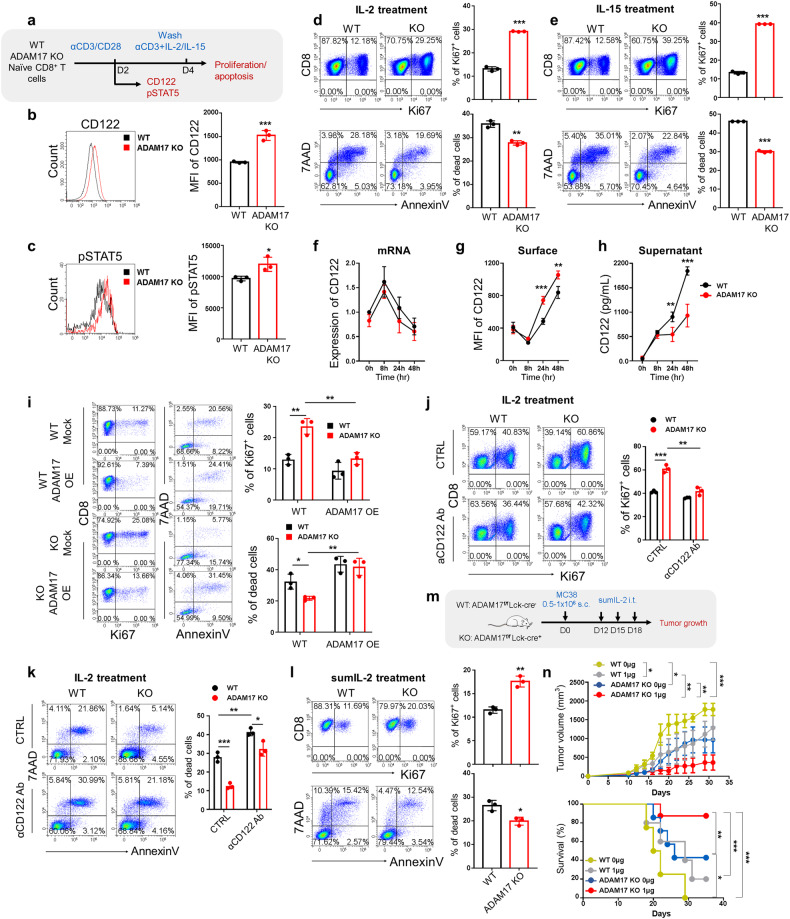
ADAM17 regulates CD8^+^ T cell response through CD122 cleavage. **a** Schematic diagram depicting the experimental design of the in vitro stimulation assay (*n* = 3). **b** Representative FACS histograms (left) and MFI level (right) of CD122 expression in WT and ADAM17 KO CD8^+^ T cells after stimulation with anti-CD3/CD28 antibodies for 2 days. **c** Representative FACS plots (left) and MFI level (right) of pSTAT5 expression in WT and ADAM17 KO CD8^+^ T cells after stimulation with anti-CD3/CD28 antibodies for 2 days followed by IL-2 re-stimulation. **d**–**e** WT and ADAM17 KO CD8^+^ T cells were stimulated with anti-CD3/CD28 antibodies for 2 days and switched into culture containing anti-CD3 antibody and cytokines IL-2 or IL-15. Representative FACS plots and the frequencies of Ki67 expression (up), Annexin V and 7AAD expression (bottom) in WT and ADAM17 KO CD8^+^ T cells in the presence of IL-2 (**d**) or IL-15 (**e**). **f**–**h** WT and ADAM17 KO CD8^+^ T cells were stimulated with anti-CD3/CD28 antibodies for different periods of time, and the CD122 expression at mRNA and protein levels were detected (*n* = 4). **f** The mRNA expression of CD122 in WT and KO CD8^+^ T cells was measured by qPCR. **g** The surface CD122 expression in WT and KO CD8^+^ T cells was measured by flow cytometry. (**h**) The levels of soluble CD122 in the culture supernatants of WT and KO CD8^+^ T cells were detected by ELISA. **i** WT and ADAM17 KO CD8^+^ T cells were stimulated with anti-CD3/CD28 antibodies for 16 hr before transfection with either mock or *Adam17*-overexpressing (OE) retrovirus for 2 days, and switched into culture containing anti-CD3 antibody and cytokines IL-2 for another 2 days. Representative FACS plots and the frequencies of Ki67 expression (left), Annexin V and 7AAD expression (right) in WT and ADAM17 KO CD8^+^ T cells. **j**, **k** In the experimental settings in (**d**), WT and ADAM17 KO CD8^+^ T cells were stimulated in the presence of IL-2 and anti-CD122 blocking antibodies. Representative FACS plots and the frequencies of Ki67 expression (**j**), and Annexin V and 7AAD expression (**k**) were shown in WT and ADAM17 KO CD8^+^ T cells. **l** WT and ADAM17 KO CD8^+^ T cells were stimulated with anti-CD3/CD28 antibodies for 2 days and switched into culture containing anti-CD3 antibody and cytokine sumIL-2. Representative FACS plots and the frequencies of Ki67 expression (up), Annexin V and 7AAD expression (bottom) in WT and ADAM17 KO CD8^+^ T cells in the presence of sumIL-2. **m** Schematic diagram showing the experimental design of the direct tumor model with sumIL-2 administration. **n** Tumor growth and survival of tumor-bearing mice were monitored over time (*n* = 4-8). Data are representative of three independent experiments shown as the mean ± SD. Statistical testing is depicted as two-sided, unpaired t-tests; *P ≤ 0.05, **P ≤ 0.01, ***P ≤ 0.001

To investigate the effects of ADAM17 on CD8^+^ T cells through CD122 signaling, we compared the CD8^+^ T cell responses to cytokines IL-2 and IL-15 upon TCR stimulation in vitro. Naïve CD8^+^ T cells from WT and ADAM17 KO (ADAM17^fl/fl^Lck-Cre^+^) mice were isolated and stimulated with anti-CD3/CD28 antibodies for two days before measurements of CD122 expression and downstream signaling (Fig. 5a). The results demonstrated that both the expression of surface CD122 (Fig. 5b) and the phosphorylation of STAT5 following IL-2 stimulation (Fig. 5c) were augmented in ADAM17-depleted CD8^+^ T cells. After two days stimulation, activated CD8^+^ T cells were then switched into culture medium containing anti-CD3 antibody and cytokines IL-2 or IL-15 for another two days and examined for cell proliferation and apoptosis (Fig. 5a). As expected, increased CD122 expression in ADAM17 KO CD8^+^ T cells led to increased cell proliferation as well as decreased apoptosis in response to both IL-2 (Fig. 5d, supplementary Fig. 7a and b) and IL-15 (Fig. 5e, supplementary Fig. 7c and d). Previously, it has been shown that CD122 was shed from the cell membrane of CD4^+^ T_reg_ cells by ADAM10.^53^ To explore whether CD122 can be cleaved in CD8^+^ T cells by ADAM17, we evaluated the soluble CD122 in the supernatant. First, the mRNA level of CD122 was comparable between WT and ADAM17 KO CD8^+^ T cells stimulated with anti-CD3/CD28 antibodies for different periods (Fig. 5f), which was in agreement with the RNA-seq data (*Il2rb*) (supplementary Fig. 6a). However, the surface CD122 expression was significantly increased in ADAM17 KO CD8^+^ T cells (Fig. 5g and Fig. 5b). Importantly, the reduced proteolytic cleavage led to the elevated surface CD122, as the soluble CD122 was remarkably decreased in the culture supernatants after ADAM17 ablation (Fig. 5h). These data strongly indicate that ADAM17 mediates the ectodomain shedding of CD122 in the effector CD8^+^ T cells. Moreover, to provide further evidence of ADAM17-mediated CD122 cleavage promoting CD8^+^ T cell survival, we overexpressed ADAM17 in WT and ADAM17 KO CD8^+^ T cells to evaluate cell proliferation and apoptosis. The results showed that ADAM17 overexpression significantly inhibited the upregulated CD122 expression (supplementary Fig. 7e) and cell proliferation (Fig. 5i and supplementary Fig. 7f) induced by ADAM17 deletion, as well as elevated cell apoptosis (Fig. 5i) in ADAM17 KO CD8^+^ T cells.

The substrate selectivity of ADAM17 is regulated by a variety of mechanisms, involving its structural domains. ADAM17 is a multi-domain protein containing a N-terminal signal sequence, followed by a prodomain (PD), a catalytic metalloprotease domain (MD), a disintegrin domain (DD), a membrane-proximal protein domain (MPD), a conserved ADAM17 interaction sequence (CANDIS), a transmembrane domain (TM), and a C-terminal cytoplasmic domain (CD)^26^ (supplementary Fig. 7g). Previous studies have reported that the non-catalytic domains, such as the MPD,^54^ CANDIS,^55^ and TM,^56^ of ADAM17 play critical roles in regulating its substrate recognition. To study the mechanisms of ADAM17 cleaving CD122, we constructed ADAM17 variants with mutations in the MPD (mutant 1: R625G/K626G/K628G), CANDIS (mutant 2: L659P) and TM (mutant 3: F687A/S688A) domains, respectively (supplementary Fig. 7h). By transient transfection of expression vectors encoding CD122 together with either WT or mutant ADAM17 into HEK293 cells (supplementary Fig. 7i), the initial attempt has identified that the MPD (mutant 1) and TM (mutant 3) domains of ADAM17 were essential for CD122 cleavage (supplementary Fig. 7j and k).

To provide further evidence of ADAM17-mediated CD122 shedding in enhancing CD8^+^ T cell survival, we evaluated the cell proliferation and apoptosis in WT and ADAM17 KO CD8^+^ T cells stimulated in vitro in the presence of anti-CD122 blocking antibodies. As expected, CD122 blockade remarkably impaired the enhanced cell survival in ADAM17 KO CD8^+^ T cells treated with IL-2 (Fig. 5j, k and supplementary Fig. 8a). As a pivotal cytokine for effector CD8^+^ T cells growth and cytotoxic functions, IL-2, however, has not been successful in cancer immunotherapy due to the fact that IL-2 is predominantly consumed by T_reg_ cells which had high expression of the high-affinity IL-2Rα, CD25.^57,58^ Strategies of developing IL-2 mutations (IL-2 muteins) which have increased affinities for CD122 binding can preferentially expand CD8^+^ T cells and have exhibited enhanced cancer therapeutic effects.^59–62^ Thus, to further verified the effects of CD122 pathway, we also applied a super mutant IL-2 (sumIL-2) which is engineered to have decreased CD25 affinity but increased CD122 binding.^59^ Compared to WT counterparts, ADAM17 deficient CD8^+^ T cells also displayed significantly increased cell survival after sumIL-2 treatment (Fig. 5l, supplementary Fig. 8b and c), strongly indicating that CD122 signaling was enhanced in CD8^+^ T cells after ADAM17 ablation. Previous work has demonstrated that local injection of sumIL-2 effectively controlled the tumor growth while limit systemic toxicity.^59^ Therefore, we also revealed that ADAM17 deficiency exhibited a synergistic anti-tumor effect when combined with sumIL-2 treatment. WT (ADAM17^fl/fl^Lck-Cre^-^) and ADAM17 KO (ADAM17^fl/fl^Lck-Cre^+^) mice were inoculated with colon cancer cells MC38 followed by intratumoral injection of sumIL-2 (Fig. 5m). In agreement with the results from melanoma tumor model (Fig. 3b), ADAM17 deficiency in T cells showed a superior anti-tumor activity than WT controls (Fig. 5n and supplementary Fig. 8d), which also demonstrated an intrinsic role of ADAM17 in CD8^+^ T cell function. While intratumoral administration of low-dose sumIL-2 alone could effectively control the tumor growth, the combination of sumIL-2 treatment with ADAM17 ablation in T cells further enhanced the antitumor efficacy (Fig. 5n and supplementary Fig. 8d). These data suggest that CD122 is regulated post-translationally by ADAM17 and thus, ADAM17 deletion promotes CD8^+^ T cell survival and effector function through enhancing CD122 signaling.

### Targeting ADAM17 improves the anti-tumor effects of CAR-T cell therapy

**Fig. 6:**
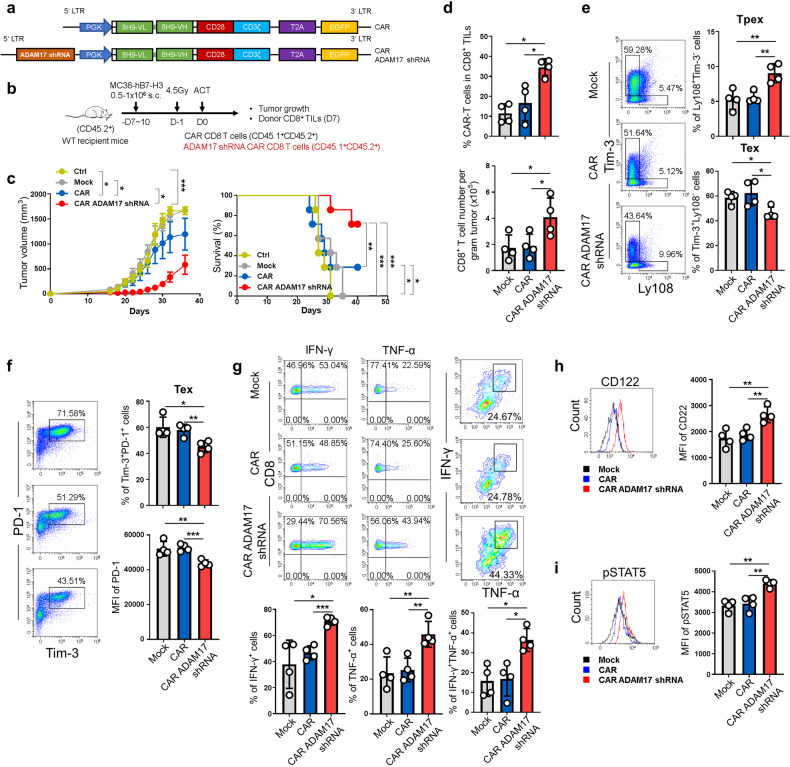
Targeting ADAM17 enhances CAR-T cells efficacy in tumor eradication. **a** Schematic structures of anti-hB7-H3 (8H9) CAR and anti-hB7-H3 CAR with an *Adam17* shRNA. **b** Schematic diagram depicting the experimental design of the mouse CAR-T tumor model without IL-2/sumIL-2 treatment. **c** Tumor growth and survival of tumor-bearing mice were monitored over time (n = 7). **d** Percentage of transferred CAR-T cells in CD8^+^ TILs with indicated gene modifications in tumor-infiltrating immune cells (up) and the absolute cell number of CAR-T cells per gram tumor tissue in each group (bottom) (*n* = 4). **e** Representative FACS plots of Tim-3 and Ly108 expression in different groups of tumor-infiltrating CAR-T cells (left). The frequencies of Ly108^+^Tim-3^-^ and Tim-3^+^Ly108^-^ cells in CAR-T cells (right). **f** Representative FACS plots of PD-1 and Tim-3 expression in CAR-T cells (left). The frequencies of Tim-3^+^PD-1^+^ cells and the MFI of PD-1 in different groups of CAR-T cells (right). **g** Representative FACS plots of IFN-γ and TNF-α production in CAR-T cells (up). The frequencies of IFN-γ^+^, TNF-α^+^ and IFN-γ^+^TNF-α^+^ cells in CAR-T cells (bottom). **h** Representative FACS histograms (left) and MFI level (right) of CD122 expression in CAR-T cells isolated from the tumor. **i** Representative FACS plots (left) and MFI level (right) of pSTAT5 expression in CAR-T cells isolated from tumor tissues and stimulated ex vivo with IL-2. Data are representative of three independent experiments shown as the mean ± SD. Statistical testing is depicted as two-sided, unpaired t-tests; *P ≤ 0.05, **P ≤ 0.01, ***P ≤ 0.001

Intrigued by the role of ADAM17 in modulating the anti-tumor reactivity of CD8^+^ T cells, we next explored the application of inhibiting ADAM17 in CAR-T cell therapy. To this end, we developed mouse CAR-T cells with ADAM17 inhibition based on a second-generation CAR that specifically targets human tumor antigen B7-H3 (hB7-H3)^63^ (Fig. 6a). CD8^+^ T cells from WT mice were transduced with either anti-hB7-H3 CAR or anti-hB7-H3 CAR expressing an shRNA against *Adam17*, and adoptively transferred into tumor-bearing mice with MC38 expressing hB7-H3, without additional IL-2/sumIL-2 treatment (Fig. 6b). Transduction of Adam17 shRNA efficiently downregulated ADAM17 expression (supplementary Fig. 8e). Compared to control groups with either no T cell transfer (Ctrl) or T cells transduced with an empty vector (Mock), CAR-T cells (CAR) exhibited potent anti-tumor activity, whereas CAR-T cells engineered to knockdown ADAM17 (CAR ADAM17 shRNA) had remarkably enhanced tumor eradication efficacy (Fig. 6c and supplementary Fig. 8f). To better understand the tumor-control advantage of CAR-T cells inhibiting ADAM17, we assessed the proportions and phenotypes of these tumor-infiltrating CAR-T cells 7 days after adoptive transfer (supplementary Fig. 8g). The percentage of CD8^+^ T cells among CD45^+^ cells was increased in CAR ADAM17 shRNA group (supplementary Fig. 8h). Moreover, the frequency and cell number of ADAM17 shRNA CAR-T cells were significantly increased compared to Mock- and CAR-T cells (Fig. 6d). The frequencies of Tpex (Ly108^+^Tim-3^-^) cells and Tex (Tim-3^+^Ly108^-^ and Tim-3^+^PD-1^+^) cells in ADAM17 shRNA CAR-T cells were increased and decreased, respectively (Fig. 6e and f). The expression level of PD-1 was also reduced (Fig. 6f). In addition, ADAM17 shRNA CAR-T cells displayed significantly elevated IFN-γ^+^, TNF-α^+^, as well as IFN-γ^+^TNF-α^+^ polyfunctional CD8^+^ T cells (Fig. 6g). Consistent with ADAM17 KO mice, CAR-T cells with ADAM17 inhibition displayed increased CD122 expression in both tumor (Fig. 6h) and draining LNs (supplementary Fig. 8i), as well as STAT5 phosphorylation after IL-2 stimulation ex vivo (Fig. 6i). Collectively, we found that ADAM17 deletion can markedly improve the anti-tumor activity of both CD8^+^ TCR-T and CAR-T cells by enhancing CD122 signaling, highlighting its potential for developing next-generation cancer immunotherapies.

### ADAM17 restrains CD122 signaling in human CD8^+^ T cells

**Fig. 7:**
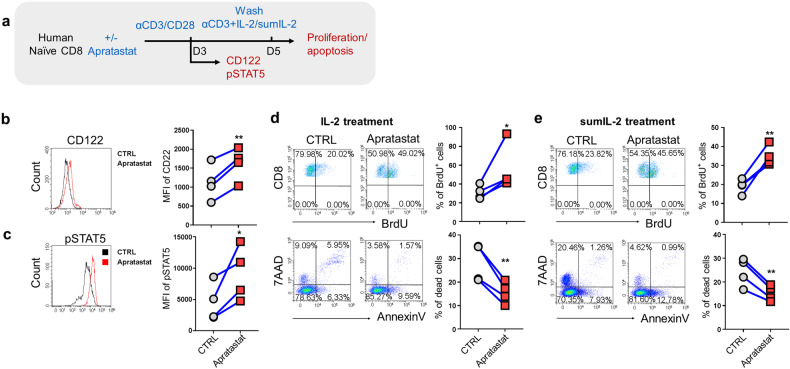
ADAM17 restrains CD122 signaling in human CD8^+^ T cells. **a** Schematic diagram depicting the experimental design of the in vitro stimulation assay (*n* = 4). **b** Representative FACS histograms (left) and MFI level (right) of CD122 expression in human CD8^+^ T cells treated with or without ADAM17 inhibitor Apratastat after stimulation with anti-CD3/CD28 antibodies for 3 days. **c** Representative FACS plots (left) and MFI level (right) of pSTAT5 expression in human CD8^+^ T cells treated with or without ADAM17 inhibitor Apratastat after stimulation with anti-CD3/CD28 antibodies for 3 days followed by IL-2 re-stimulation. **d**–**e** Human CD8^+^ T cells with or without Apratastat treatment were stimulated with anti-CD3/CD28 antibodies for 3 days and switched into culture containing anti-CD3 antibody and cytokines IL-2 or sumIL-2. Representative FACS plots and the frequencies of BrdU incorporation (up), Annexin V and 7AAD expression (bottom) in human CD8^+^ T cells in the presence of IL-2 (**d**) or sumIL-2 (**e**). Data are shown as the mean ± SD. Statistical testing is depicted as two-sided, unpaired t-tests; *P ≤ 0.05, **P ≤ 0.01

To further explore whether the regulation of CD122 by ADAM17 exists in human CD8^+^ T cells, we applied a potent and selective ADAM17 inhibitor, Apratastat,^25^ in human CD8^+^ T cells upon TCR stimulation in vitro. Naïve (CD45RA^+^CD45RO^-^) CD8^+^ T cells from the peripheral blood mononuclear cells (PBMCs) of healthy donors were isolated by flow cytometry and stimulated with anti-CD3/CD28 antibodies in the presence of Apratastat for three days before detecting CD122 expression and STAT5 phosphorylation (Fig. 7a). In consistent with results in mouse CD8^+^ T cells (Fig. 5), both the CD122 expression (Fig. 7b) and the phosphorylation of STAT5 following IL-2 re-stimulation (Fig. 7c) were elevated in human CD8^+^ T cells after ADAM17 inhibition. Further, activated CD8^+^ T cells were then switched into a culture medium containing anti-CD3 antibody and cytokines IL-2 or sumIL-2 for another two days and examined for cell proliferation and apoptosis (Fig. 7a). The results showed that Apratastat-treated human CD8^+^ T cells displayed both increased cell proliferation as well as decreased apoptosis in response to both IL-2 (Fig. 7d) and sumIL-2 (Fig. 7e). These data demonstrated that the post-translational regulation of CD122 by ADAM17 is also present in human CD8^+^ T cells, which shed light on the potential application of ADAM17 manipulation for clinical cancer immunotherapy.

## Discussion

CD8^+^ T cell differentiation and functionality are regulated at transcriptional and post-transcriptional levels are extensively studied. However, the post-translational regulation, especially ADAM-mediated ectodomain shedding of membrane proteins, in CD8^+^ T cells are largely unknown. Here, we defined a critical post-translational regulatory mechanism mediated by ectodomain sheddase ADAM17 which plays a negative role in effector differentiation and function of CD8^+^ T cells during immune response in both mouse and human. T cell-specific deficiency in ADAM17 led to increased effector CD8^+^ T cell generation in response to pathogenic infection. Mechanistically, we found an elevated surface CD122 expression in ADAM17 KO CD8^+^ T cells, which enhanced their response to IL-2 and IL-15 stimulation. Hence, ADAM17 ablation in CD8^+^ T cells improved their anti-tumor activity in both TCR-T (OT-I) and CAR-T cell models.

Generally, gene expression depends on transcription, translation as well as the turnover of both mRNA and protein levels. Though there is a substantial across-gene (total genes) correlation between their abundance, within-gene (a single gene) correlation can be variable due to complex regulations, suggesting that both mRNA and protein analysis provide insightful information.^64^ The newly identified transcriptional-translational conflict, a phenomenon of simultaneous increase of transcription, while decrease in translation, adds to the complexity of RNA-protein inconsistency.^65^ In this study, we found considerable variations between mRNA and protein levels when comparing the differentially expressed genes/proteins in KLRG1^+^ effector CD8^+^ T cells in comparison to naïve cells, suggesting post-translational regulations are involved in this process. Hence, it is important and necessary that both mRNA (transcriptomic) and protein (proteomic) analyses should be integrated to reveal the gene expression profile in CD8^+^ T cells.

ADAM17, as one of the important sheddases, plays critical roles in regulating immunological functions by proteolytically cleaving a variety of membrane proteins.^25,27,28^ Previous studies have suggested that ADAM17 promotes T cell activation and proliferation by cleavage of inhibitory molecules or TNF superfamily molecules, such as Tim-3,^52^ Lag-3,^30^ FasL,^31^ Pik3ip1,^35^ CD27^33^ and CD137.^34^ However, in this study, by using mice with T cell-specific deletion of ADAM17, we found that ADAM17 inhibited the CD8^+^ T cell immune response and function, as evidenced by increased effector CD8^+^ T cell differentiation and pathogen clearance after ADAM17 ablation. Moreover, ADAM17 deletion in CD8^+^ T cells exhibited an enhanced anti-tumor activity. The discrepancy of ADAM17 in regulating CD8^+^ T cell immune response may be attributed to different experimental settings. Though more than 90 substrates are cleaved by ADAM17, ADAM17-mediated substrate cleavage is tightly regulated, highly depending on the biological context.^66^ Hence, ADAM17 preferentially conducts certain substrate cleavage in distinct pathophysiological conditions and cell types. Notably, Link et al. reported that T cell-specific knockout of ADAM17 had no profound impacts on both T cell responses to bacterial infection.^39^ Nevertheless, despite a subtle change, the frequency of CD8^+^ T cells producing TNF-α and IFN-γ was increased in ADAM17 KO mice.^39^ Consistently, we observed similar but more prominent results using mice with T cell-specific knockout ADAM17 on the OT-I TCR transgenic background.

ADAM17 has been shown to mediate proteolytic cleavage of many substrate proteins. However, here, we only identified CD122 was upregulated in ADAM17 KO effector CD8^+^ T cells. Additionally, the expression of Lag-3 and Tim-3, two known ADAM17 substrates, was also not upregulated in ADAM17 deficient CD8^+^ T cells. It is unsurprisingly that the metalloprotease-mediated substrate cleavage is highly dependent on the biological context, such as cell types, enzymatic activity, and pathological environment.^27^ For instance, Tim-3 is shed by ADAM17 and ADAM10 in human CD14^+^ monocytes,^52^ while the cleavage of Tim-3 on CD8^+^ T cells is mainly regulated by ADAM10.^67^ In addition, the cleavage of Lag-3 was mainly studied in CD4^+^ T cells,^51^ mediated by ADAM10 constitutively and by ADAM17 upon TCR stimulation.^30^ Therefore, in certain pathophysiological conditions and cell types, ADAM17 preferentially mediates specific substrates cleavage. In this study, we demonstrated that ADAM17 predominantly modulates CD122 ectodomain shedding on effector CD8^+^ T cells during immune responses.

Taking advantage of ADAM-mediated ectodomain shedding, manipulation the cleavage of receptors, such as Lag-3, CD16a, MICA and MICB, could be exploited to fine-tune the immunological functions.^51,68,69^ Here, we found that ADAM17 knockout led to increased surface CD122 expression and CD8^+^ T cell vitality and effector function. Due to the potent ability to promote CD8^+^ T cell expansion and cytotoxic activity, engineered IL-2 with increased affinity for CD122 (IL-2Rβ) have been demonstrated effective tumor control capability and response to immune checkpoint blockade.^59,60,62,70^ Those findings intrigued us to explore the potential cancer therapeutics of targeting ADAM17 in CD8^+^ T cells. As expected, CAR-T cells engineered to knock down ADAM17 displayed superior tumor eradication efficacy with increased pro-inflammatory cytokine production and surface CD122 expression. In conclusion, we reveal an essential role of ADAM17-mediated ectodomain shedding of CD122 signaling in enhancing effector CD8^+^ T cell response and anti-tumor activity. Our findings provide new insights into understanding of post-translational regulation in CD8^+^ T cell differentiation and functionality, and highlight ADAM17 as a potential therapeutic target for the development of cancer immunotherapies.

## Materials and methods

### Mice

ADAM17^fl/fl^, Lck-Cre^+^, CD45.1^+^, and OT-1 mouse strains were purchased from The Jackson Laboratory (Bar Harbor, ME, USA). ADAM17^fl/fl^ mice were crossed with Lck-Cre and OT-1 mice to generate ADAM17^fl/fl^Lck-Cre^*+*^(OT-1^+^) (KO) and ADAM17^fl/fl^Lck-Cre^*-*^(OT-1^+^) (WT) mice. CD45.1^+^ or CD45.2^+^ mice were crossed with OT-1 mice to produce OT-1^+^CD45.1^+^ or OT-1^+^CD45.2^+^ mice. Female mice were preferentially used in this study. Mice were housed in the specific pathogen-free (SPF) conditions under the Xi’an Jiaotong University Division of Laboratory Animal Research. Experimental procedures were accorded by the Institutional Animal Care and Use Committee of Xi’an Jiaotong University, Xi’an Center for Disease Control and Suzhou Institute of Systems Medicine.

### Antibodies and flow cytometry

Single-cell samples were stained with the following anti-mouse monoclonal antibodies purchased from Biolegend (San Diego, CA, USA): CD45.1 (clone A20), CD45.2 (104), CD8A (clone 53-6.7), CD3E (145-2C11), CD44 (IM7), CD62L (MEL-14), KLRG1 (2F1/KLRG1), IL-7R (A7R34), CD122 (TM-β1), CD215 (6B4C88), CD132 (TUGm2), CD226 (10E5), PD-1 (29F.1A12), Lag-3 (C9B7W), Tim-3 (B8.2C12), Ly108 (330-AJ), CD25 (PC61), CD69 (H1.2F3), CX3CR1 (SA011F11), ICOS (15F9), T-bet (4B10), Foxp3 (MF-14), IFN-γ (XMG1.2), TNF-α (Mp6-XT22), Phospho STAT5 (Tyr694) (A17016B.Rec), Ki67 monoclonal antibody (SolA15), anti-human CD45RA (HI100) and anti-human CD45RO (UCHL1). Brefeldin A, monensin, phorbol 12-myristate 13-acetate (PMA), and Ionomycin were also obtained from Biolegend.

Markers on the cell surface were stained in FACS buffer which is PBS plus 1% FBS at 4 °C for 30 min. For intracellular staining, cells were first stimulated with PMA/Ionomycin in vitro in the presence of brefeldin A and monensin for 4 hr. Then cells were fixed and permeabilized using the Fixation/Permeabilization Solution Kit (Biolegend). For nuclear proteins staining, such as Ki67 and pSTAT5, cells were fixed and permeabilized using a Fixation/Permeabilization kit for transcription factor staining that was purchased from eBioscience (San Diego, CA, USA). For BrdU staining, cells were first stained with surface markers followed by BrdU staining (BrdU Flow Kit, BD Biosciences) according to the manufacturer’s instruction. To assess the apoptosis, cells were stained with 7-Aminoactinomycin D (7AAD)/Annexin V (BD Biosciences). Flow cytometer CytoFLEX (BECKMAN COULTER) was used to analyzed cells. Data analysis was performed by FlowJo software and CytExpert software. Naïve (CD62L^+^ CD44^-^) CD8^+^ T cells or total CD8^+^ T cells were sorted out using the FACSAria sorter (BD Biosciences).

### T cell transfer and LM-OVA infection

5×10^4^ naïve CD8^+^ T cells (OT-1^+^) were sorted out by FACSAria II (BD Bioscience) cell sorter from both WT (CD45.1^+^) and KO (CD45.2^+^, ADAM17^fl/fl^Lck-Cre^*+*^) mice and either separately transferred or co-transferred into WT recipient mice (CD45.1^+^CD45.2^+^) at a ratio of 1:1. 24 hr after the cell transfer, mice were administrated with 2×10^4^ colony-forming units (CFU) of *LM*-OVA intravenously (i.v.). T cells were examined at indicated days post-infection. To quantify bacterial burden, 1×10^6^ CFU *LM*-OVA was applied, and spleen and liver tissues were collected and homogenized in PBS. Tissue homogenate at serial dilutions were prepared and 50 μl was plated on brain-heart infusion (BHI) agar plates. After incubation overnight at 37 °C, *LM* CFUs was calculated by counting the colonies.

### Tumor model and preparation of tumor-infiltrating lymphocytes (TILs)

In the direct tumor model, ADAM17 WT (ADAM17^fl/fl^Lck-Cre^-^) and KO (ADAM17^fl/fl^Lck-Cre^+^) mice were inoculated subcutaneously (s.c.) on the right flank with 5 × 10^5^ melanoma B16-F10 cells. Tumor growth was monitored using a digital Vernier caliper every 3 days. The tumor volume was determined by using the formula V = (L × W2)/2, in which V is the tumor volume, L represents the tumor length (longer diameter) and W is the tumor width (shorter diameter). In some experiments, sumIL-2 was administrated on day 12, 15 and 18 after tumor implantation by intratumoral injection (i.t.) of 1 µg/mouse. In the adoptive cell transfer (ACT) model, WT mice were inoculated s.c. with 5×10^5^ melanoma B16-OVA cells for 7-10 days when the tumor volume reaches ~100 mm3, followed by a total body irradiation (4.5 Gy) and the adoptive transfer of OT-1^+^ CD8^+^ T cells from either WT or ADAM17 KO mice. Similarly, in the CAR-T tumor model, WT mice were inoculated s.c. with 5×10^5^ MC38-hB7-H3 cells for 7-10 days when the tumor volume reaches ~100 mm3, followed by a total body irradiation (4.5 Gy) and the adoptive transfer of different types of CAR-T cells.

Tumor tissues were excised 14 days after the implantation (direct tumor model) or 7–10 days after CD8^+^ T cell adoptive transfer (ACT and CAR-T model) for weight measurement or photography. For TILs analysis, excised tumor tissues were minced and digested with 2 mg/ml collagenase IV (LS004186, Worthington) and 25 µg/ml DNAse I (D8071, Solarbio) at 37 °C on a shaker for 1 h. The digested cells were passed through 70-μm filters to remove undigested tumor tissues or debris.

### Retroviral production and mouse CAR-T cell generation

The CAR construct used in this study was a generous gift which has been described previously.^63^ The B7-H3 CAR was incorporated with 8H9 scFv, CD28, CD3ζ intracellular signal domain, and EGFP. The ADAM17 shRNA CAR was incorporated with *Adam17* shRNA, 8H9 scFv, CD28, CD3ζ intracellular signal domain, and EGFP. CAR^+^ T cells were identified by EGFP expression. CAR retrovirus was prepared by transfecting 0.3 μg pCL-Eco retrovirus packaging vector plus 2.4 μg of each retroviral expressing vector into BOSC-23 cells cultured in 6-well plates using jetPRIME transfection reagent (Polyplus). Retrovirus in the culture supernatant was collected at both 48 hr and 72 hr through 0.45 µm filters. For in vitro T cell stimulation, purified CD8^+^ T cells were cultured overnight using 5 µg/ml plate-bound anti-CD3 and 2 µg/mL anti-CD28 antibodies. Retrovirus supernatant was added into stimulated T cells, and spin infection was performed at 37 °C at 2500 rpm for 90 min. EGFP^+^ cells were monitored by flow cytometry analysis or sorted out for further experiments. The *Adam17* shRNA sequence was CCCTTGAAGAATACTTGTAAA.

### T-cell in vitro stimulation

To examine the T cell activation, proliferation, and apoptosis in vitro, splenocytes were isolated and stimulated with 2.5 μg/ml coated anti-CD3 and 1 μg/ml soluble anti-CD28 antibodies for indicated time points (activation), 72 hr (proliferation) or 48 hr (apoptosis). To evaluate CD8^+^ T cell response to cytokines IL-2 and IL-15, naïve CD8^+^ T cells were stimulated in vitro with 2.5 μg/ml coated anti-CD3 and 1 μg/ml soluble anti-CD28 antibodies for 48 hr. Activated CD8^+^ T cells were then washed and switched to stimulation containing 2.5 μg/ml coated anti-CD3 antibody and 2.5 ng/ml IL-2 or sumIL-2 or 50 ng/ml IL-15 for another two days. In some assays, 10 μg/ml anti-CD122 blocking antibodies (clone TM-β1, Biolegend) were added. For detecting pSTAT5, activated CD8^+^ T cells were washed and starved in culture medium without FBS for 1 hr before adding 2.5 ng/ml IL-2 for 10 min. To detect ADAM17 substrate on the surface, splenocytes from OT-1^+^ WT and ADAM17 KO mice were stimulated with 1 µg/ml OVA peptide for the indicated time, surface CD62L and TNF-α were examined by flow cytometry. To evaluate the CD122 cleavage, naïve CD8^+^ T cells from WT and ADAM17 KO mice were stimulated in vitro with 2.5 μg/ml coated anti-CD3 and 1 μg/ml soluble anti-CD28 antibodies. Cells and culture supernatants were collected at 0 hr, 8 hr, 24 h and 48 hr after the stimulation, and subjected to expression measurement at the mRNA and protein levels. For human CD8^+^ T cells, naïve (CD45RA^+^CD45RO^-^) CD8^+^ T cells were sorted by flow cytometry from healthy human PBMCs and stimulated in vitro with 5 μg/ml coated anti-CD3 and 2 μg/ml soluble anti-CD28 antibodies with or without 1 uM Apratastat (MedChemExpress) treatment for 72 hr. Activated CD8^+^ T cells were then washed and switched to stimulation containing 5 μg/ml coated anti-CD3 antibody and 2.5 ng/ml IL-2 or sumIL-2 for another two days.

### cDNA constructs and transfection

Expression plasmids of mouse CD122 WT, ADAM17 WT, and mutants (mutant 1: R625G/K626G/K628G; mutant 2: L659P; mutant 3: F687A/S688A) were constructed into MSCV-based expressing vectors. Mutations were introduced via site-directed mutagenesis PCR. To analyze the enzymatic activity of ADAM17, HEK293 cells cultivated in Dulbecco’s Modified Eagle’s medium (DMEM, Gibco) were transfected with 2.4 µg each expression plasmid of WT CD122 and different ADAM17 variants using jetPRIME transfection reagent (Polyplus). Cells were harvested 48 hr after transfection for expression measurement.

### Enzyme-linked immunosorbent assay (ELISA)

Supernatants from T cell cultures were collected and measured for soluble CD122 using Mouse IL-2sRβ/CD122 (Soluble Interleukin-2 Receptor Beta Chain) ELISA Kit (FineTest) according to the manufacturer’s protocol (detection range 62.5-4000 pg/ml; sensitivity: < 37.5 pg/ml).

### Statistical analysis

All data were shown as means ± SD/SEM, number per group and number of experimental replicates were indicated in the figure legends. Statistical calculations were performed using two-tailed Student’s t-test in GraphPad Prism 9 software. The distribution of the data was shown as bar graphs with means and error bars. Statistical significance is interpreted as * *P* < 0.05, ** *P* < 0.01, *** *P* < 0.001.

### Supplementary information

supplemental method and figures

Original western blot figures

Dataset 1

Dataset 2

Dataset 3

Dataset 4

Dataset 5

Dataset 6

Dataset 7

## Data Availability

The RNA-seq data generated in this study have been deposited in the Gene Expression Omnibus (GEO) archive under accession GSE236582.
