# Supplementary material for: Targeting a disintegrin and metalloprotease (ADAM) 17-CD122 axis enhances CD8+ T cell effector differentiation and anti-tumor immunity
Source: Signal Transduct Target Ther. 2024 Jun 26;9:152. doi: 10.1038/s41392-024-01873-6 (PMC11199508; doi:10.1038/s41392-024-01873-6)
Supplement: Supplementary file 1 — supplemental method and figures [file 41392_2024_1873_MOESM1_ESM.docx]

Supplementary Materials for

Targeting A Disintegrin and Metalloprotease (ADAM) 17-CD122 axis enhances CD8^+^ T cell effector differentiation and anti-tumor immunity

Lina Sun, Anjun Jiao, Haiyan Liu, Renyi Ding, Ning Yuan, Biao Yang, Cangang Zhang, Xiaoxuan Jia, Gang Wang, Yanhong Su, Dan Zhang, Lin Shi, Chenming Sun, Aijun Zhang, Lianjun Zhang and Baojun Zhang

Correspondence to: cm.sun@xjtu.edu.cn, [zhangaijun@sdu.edu.cn](mailto:zhangaijun@sdu.edu.cn), [zlj@ism.cams.cn](mailto:zlj@ism.cams.cn), bj.zhang@mail.xjtu.edu.cn

**This PDF file includes:**

Materials and Methods

Figures. S1 to S8

Materials and Methods

*Quantitative PCR (qPCR)*

Total RNA from purified T cells was extracted using the RNeasy Mini Kit (Qiagen) following the manufacturer’s protocol. cDNA library was prepared by reverse transcription using an cDNA synthesis kit (TOYOBO) and further amplified by SYBR Green RT-qPCR Mastermix (GenStar) at StepOnePlusTM Real-Time PCR System (ThermoFisher). qPCR primers used in this study were: mouse ADAM17 5’-CGGAGGAAGCAGGCTCTG-3’, 5’- GTTTCTAAGTGTGTCGCAGACTG-3’; mouse CD122 5’- CTCAAGTGCCACATCCCAGATC-3’, 5’- AGCACTTCCAGCGGAGAGATCT-3’; and mouse β-Actin 5’- ATCTGGCACCACACCTTCTACA-3’, 5’- ACGTACATGGCTGGGGTGTT-3’.

*Western blotting*

To extract total proteins, CD8^+^ T cells at indicated experiments were lysed in RIPA lysis buffer (Beyotime Biotechnology). Protein samples were loaded and separated on SDS-polyacrylamide gels and electro-transferred onto Polyvinylidene fluoride (PVDF) membranes (Millipore). Membranes were blocked with 5% skimmed milk, incubated with indicated primary antibodies at 4°C overnight, followed by incubation with secondary antibodies at room temperature for one hour. The protein expression was measured by Fusion-Solo.6s (VILBER). Primary antibodies used in Western blot assay were: p-ERK1/2 (p-p44/42) (clone D13.14.4E, CST), ERK1/2 (clone MK1, Santa Cruz), p-p65 (clone 27.Ser 536, Santa Cruz), p65 (clone E379, Abcam), caspase 3 (clone D3R6Y, CST), ADAM17 (clone JM10-35, Invitrogen) and Actin (clone 7D2C10, Proteintech). Secondary antibodies purchased from Cwbio were: HRP goat anti-rabbit IgG and HRP goat anti-mouse IgG.

*Protein purification and mass spectrometry analysis*

Naïve and KLRG1^+^CD127^-^ CD8^+^ SLECs were sorted from WT mice and from the *in vivo* adoptive transfer model after *LM-OVA* infection on day 7, respectively. In addition, in the co-transfer model, WT and ADAM17 KO donor-derived CD8^+^ T cells were sorted out from the spleens in the recipient mice 7 days post *LM*-OVA infection. Cells was subjected to a three-time sonication using a ultrasonic processor (Scientz) in the lysis buffer (8 M urea containing 1% protease inhibitor cocktail (Merck Millipore)) on ice. Membrane protein enrichment was performed by using Mem-PER™ Plus Membrane Protein Extraction Kit (Thermo Scientific) according to the manufacturer’s protocol. For trypsin digestion, the protein solution was reduced with 5 mM dithiothreitol at 56 °C for 30 min, then alkylated in 11 mM iodoacetamide for 15 min at room temperature in darkness. First trypsin digestion was performed by adding trypsin (Promega) at a 1:50 trypsin-to-protein mass ratio and digesting overnight, and the second digestion was using a 1:100 trypsin-to-protein mass ratio for 4hr. The digested peptides were further desalted by the C18 SPE column.

For LC-MS/MS, the tryptic peptides were firstly dissolved in solvent A (0.1% formic acid and 2% acetonitrile/in water), and next directly loaded onto a reversed-phase analytical column which has 25-cm length and 75/100 μm i.d.. Peptides were further separated with a gradient (from 6% to 24%) in solvent B (0.1% formic acid in acetonitrile) over a 70-min process, then 24% to 35% in 14 min and climbing up to 80% in 3 min, followed by last 3-min holding at 80%, all of which were at a constant flow rate of 450 nl/min using the nanoElute UHPLC system (Bruker Daltonics). The peptides were further subjected to a capillary source and the timsTOF Pro mass spectrometry (Bruker Daltonics). The applied electrospray voltage was 1.60 kV. Precursors and fragments were analyzed by a TOF detector, with a setting of the MS/MS scan range from 100 to 1700 m/z. The timsTOF Pro was operated in parallel accumulation serial fragmentation (PASEF) mode. Precursors with the charge states 0 to 5 were selected for further fragmentation, as well as 10 PASEF-MS/MS scans were acquired per cycle. The dynamic exclusion was set as 30 sec.

For data processing, the resulting MS/MS data were processed by MaxQuant search engine (v.1.6.15.0). Tandem mass spectra were searched against the mouse protein expression database—Mus_musculus_10090_SP_20230103.fasta database which contains a total of 17,132 entries. Trypsin/P was selected as the cleavage enzyme allowing up to 2 missing cleavages. The mass tolerance for precursor ions was set as 20 ppm in the first search and 5 ppm in the main search, whereas the mass tolerance for fragment ions was set as 0.02 Da. Carbamidomethyl on Cys was specified as a fixed modification, acetylation on the N-terminal of a protein, and the oxidation on Met were set as variable modifications. FDR was adjusted to < 1%. Differential expressed protein was identified as P value < 0.05 and fold-change > 1.5.

*RNA-Seq library preparation and sequencing*

In the co-transfer model, donor-derived CD8^+^ T cells were sorted from the recipient mice from the spleens 7 days after *LM*-OVA infection. Total RNA was prepared with the RNeasy Mini Kit (Qiagen) following the manufacturer’s instruction. RNA concentration was assessed using the Qubit Fluorometer (Invitrogen) through the Qubit RNA broad range assay. RNA quality was detected using Agilent 2100 (Agilent Technologies, Palo Alto, CA, USA) and an RNase-free agarose gel. 200 ng total RNA was used to generated RNA-Seq libraries using TruSeq RNA sample prep kit (Illumina). mRNAs were enriched by Oligo(dT) and fragmented with fragmentation buffer randomly, followed by the cDNA synthesis. After terminal repair, cDNA library was prepared by PCR enrichment and size selection. Next, the cDNA library was subjected to sequencing by the Illumina Hiseq 2000 sequencer (Illumina HiSeq 2000 v4 Single-Read 50 bp) after pooling based on its expected data volume and effective concentration. The raw reads were firstly trimmed using the Trim Galore (version 0.5.0_dev, Cutadapt version 1.15) with default parameters. After removing those low-quality bases RNAs, clean data were mapped to mouse genome GRCm38 using Tophat2 RNA-Seq alignment software; unique reads were retained to calculate gene expression counts from Tophat2 alignment files. R environment was applied for data analysis and processing. Analysis of differential gene expression was performed using R package DESeq2 with adjusted P value < 0.05 and log_2_ fold-change greater than 1. Heat maps and bar graphs were visualized through the R pack­age.

*Bioinformatic analysis*

The functional annotations of the differentially expressed genes (DEGs) from RNA-seq data and differentially expressed proteins (DEPs) from MS analysis were performed. Gene Ontology (GO) analysis of DEGs was performed using clusterProfiler (V3.18) package. The UniProt-GOA database (<http://www.ebi.ac.uk/GOA/>) was selected to perform the GO annotation of proteome. Protein IDs identified were converted to the UniProt IDs for mapping to GO IDs. Proteins classification by GO annotation was based on the following three categories: biological process (BP), cellular component (CC) and molecular function (MF). Kyoto Encyclopedia of Genes and Genomes (KEGG) pathway enrichment analysis was conducted by KAAS (KEGG Automatic Annotation Server), a KEGG online service tools, for functional annotation of genes and KEGG Mapper pathway mapping. Gene Set Enrichment Analysis (GSEA) analysis of DEGs and DEPs were performed by the scaled gene expression matrix and GSEA package (V.4.1) which were available at the Molecular Signatures Database (MSigDB, https://www.gseamsigdb.org/gsea/downloads.jsp) with either the immunologic signature gene sets (C7) or Hallmark Gene Sets.

Supplementary figures


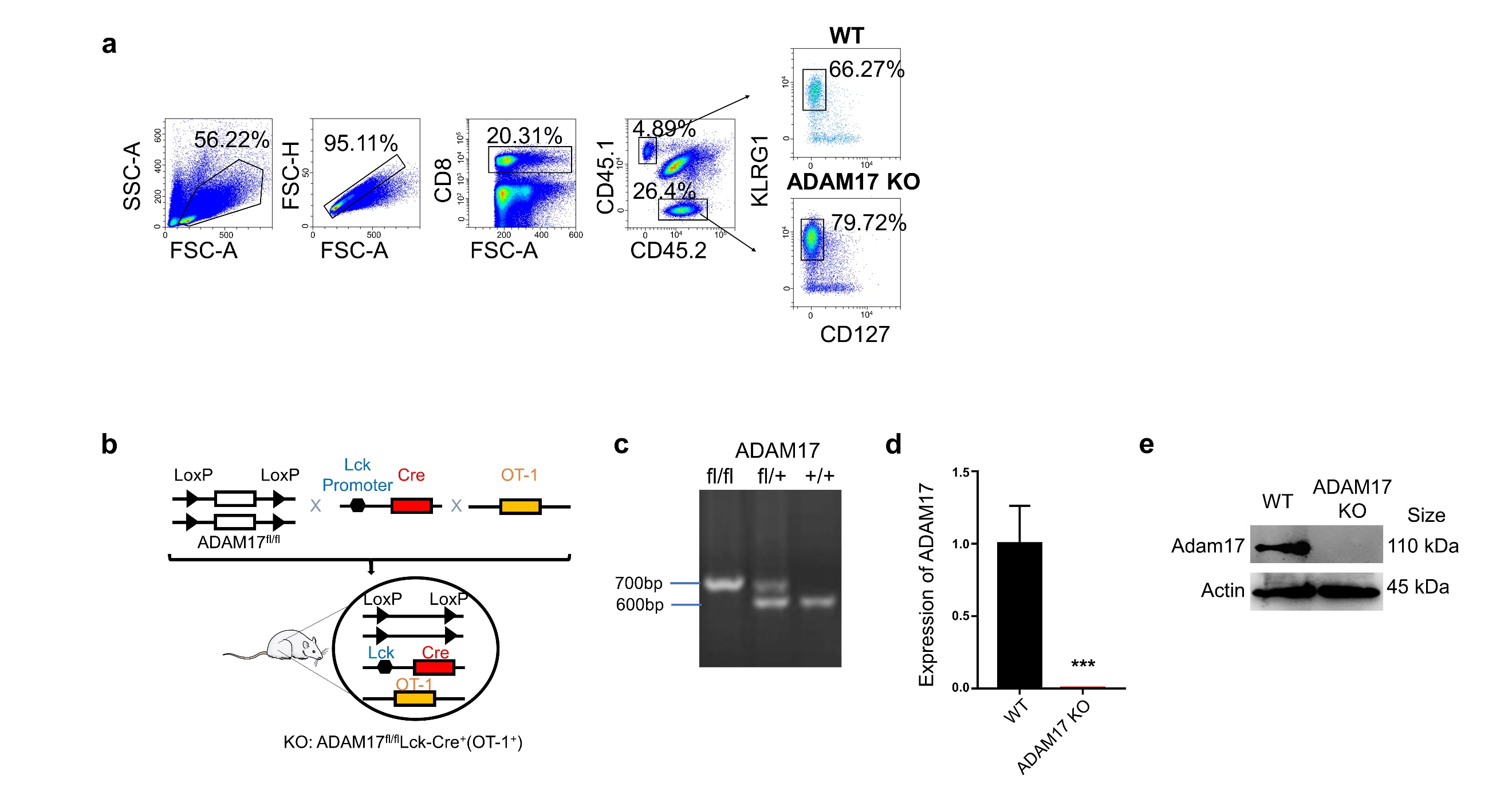


**Figure. S1.**

**ADAM17 is depleted in CD8^+^ T cells from conditional knockout mice.** (**a**) Gating strategy showing that the KLRG1^+^CD127^-^ SLECs in donor-derived CD8^+^ T cells from WT (CD45.1^+^) and ADAM17 KO (CD45.2^+^) mice (*ADAM17^fl/fl^Lck-Cre^+^*) in the *in vivo* acute infection model were isolated and subjected to RNA-seq and MS-based proteomics, respectively. (**b**) The strategy for generating CD8-specific ADAM17 deficient mice. *ADAM17* flox mice were crossed with Lck-Cre transgenic strain to generate T cell-specific *ADAM17* knock-out mice (*ADAM17^fl/fl^Lck-Cre^+^*), which were further crossed with OT-1 transgenic mice. (**c**) Genotyping of WT and *ADAM17^fl/fl^* mice by agarose gel electrophoresis of PCR. (**d**) The mRNA level of ADAM17 in CD8^+^ T cells from WT and KO (*ADAM17^fl/fl^Lck-Cre^+^*) mice was measured by qPCR (n=3). (**e**) The protein expression of ADAM17 in WT and KO (*ADAM17^fl/fl^Lck-Cre^+^*) CD8^+^ T cells after *in vitro* stimulation with anti-CD3/CD28 antibodies for 48hrs was measured by Western blot. Data are shown as the mean ± SD. Statistical testing is depicted as two-sided, unpaired t-tests; ***P ≤ 0.001.


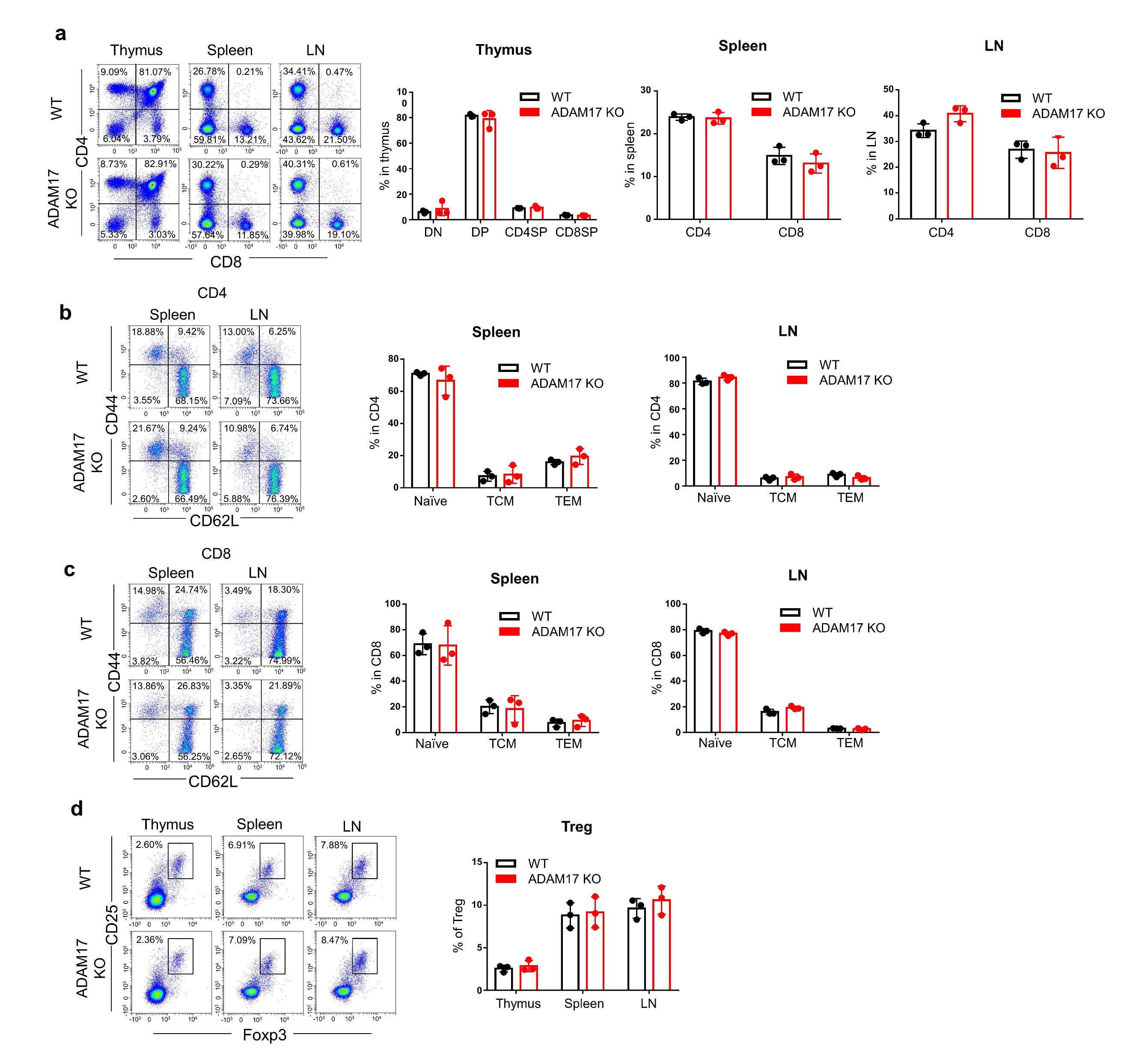


**Figure. S2.**

**ADAM17 deficiency does not affect T cell development.** (**a**) The representative FACS plots and percentages of CD4^+^ and CD8^+^ T cells in the thymus, spleen and LN in ADAM17 WT (*ADAM17^fl/fl^Lck-Cre^-^*) and KO (*ADAM17^fl/fl^Lck-Cre^+^*) mice (n=3). (**b**) The representative FACS plots and percentages of naïve (CD62L^+^CD44^-^), TCM (CD62L^+^CD44^+^) and TEM (CD62L^-^CD44^+^) CD4^+^ T cells in the spleen and LN in WT and KO mice. (**c**) The representative FACS plots and percentages of naïve (CD62L^+^CD44^-^), TCM (CD62L^+^CD44^+^) and TEM (CD62L^-^CD44^+^) CD8^+^ T cells in the spleen and LN in WT and KO mice. (**d**) The representative FACS plots and percentages of T_reg_ (CD4^+^CD25^+^Foxp3^+^) cells in the thymus, spleen and LN in WT and KO mice. Data are shown as the mean ± SD.


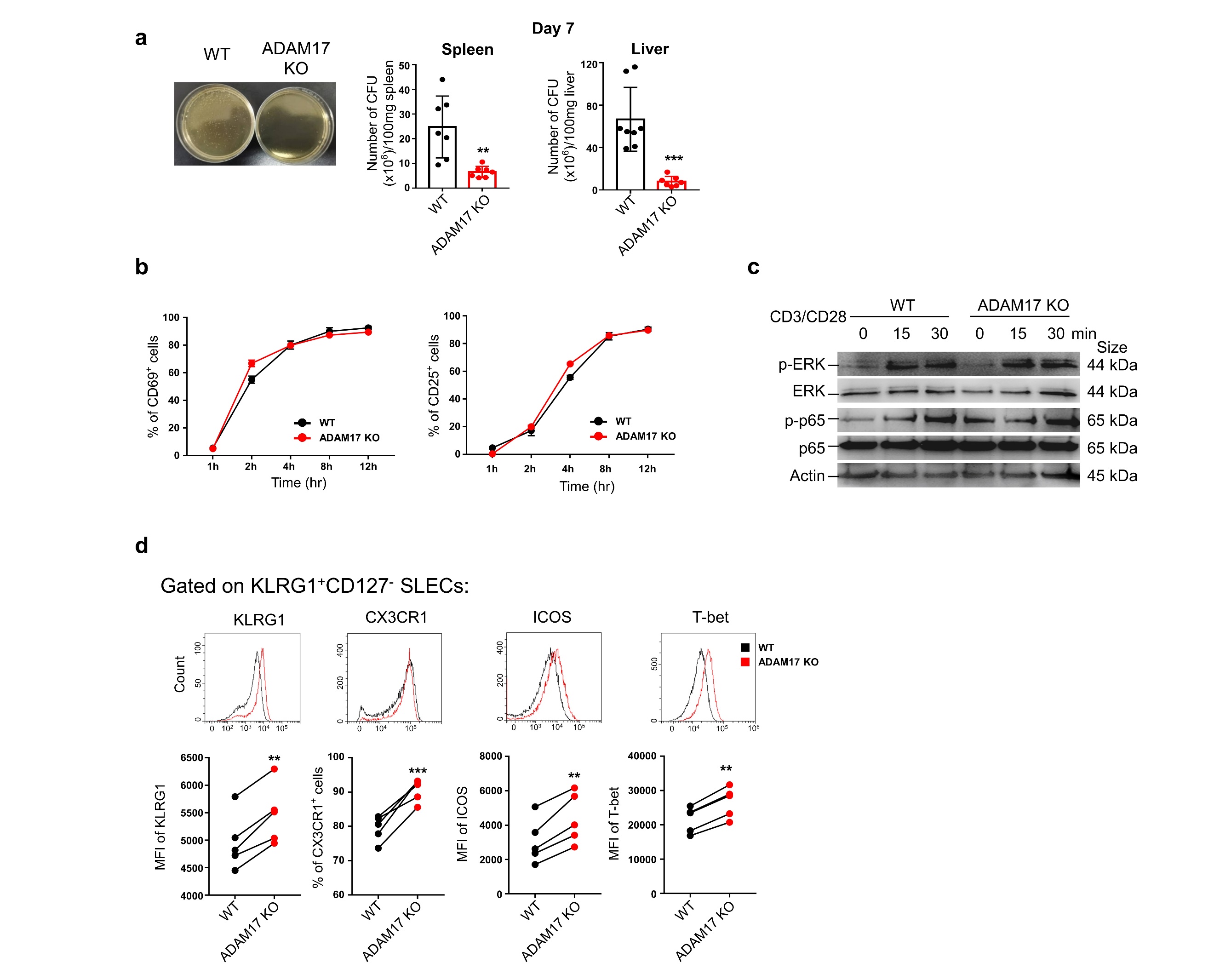


**Figure. S3.**

**Immune responses of ADAM17 deficient CD8^+^ T cells.** (**a**) Bacterial loads were determined 7 days after infection in the spleen and liver as indicated in **Fig. 1c** (n=8). (**b**) The time kinetics of CD69 (left) and CD25 (right) expression in WT and ADAM17 KO CD8^+^ T cells stimulated *in vitro* with anti-CD3/CD28 antibodies for the indicated time (n=3). (**c**) WT and ADAM17 KO CD8^+^ T cells were stimulated *in vitro* with anti-CD3/CD28 antibodies for 0, 15 and 30 min. The protein expression of p-ERK, ERK, p-p65 and p65 were measured by Western blot. (**d**) Representative FACS plots (up) and MFI (bottom) of effector markers KLRG1, CX3CR1, ICOS and T-bet in WT and ADAM17 KO KLRG1^+^CD127^-^ SLECs from the *in vivo* acute infection model. Data are shown as the mean ± SD. Statistical testing is depicted as two-sided, unpaired t-tests; **P ≤ 0.01, ***P ≤ 0.001.


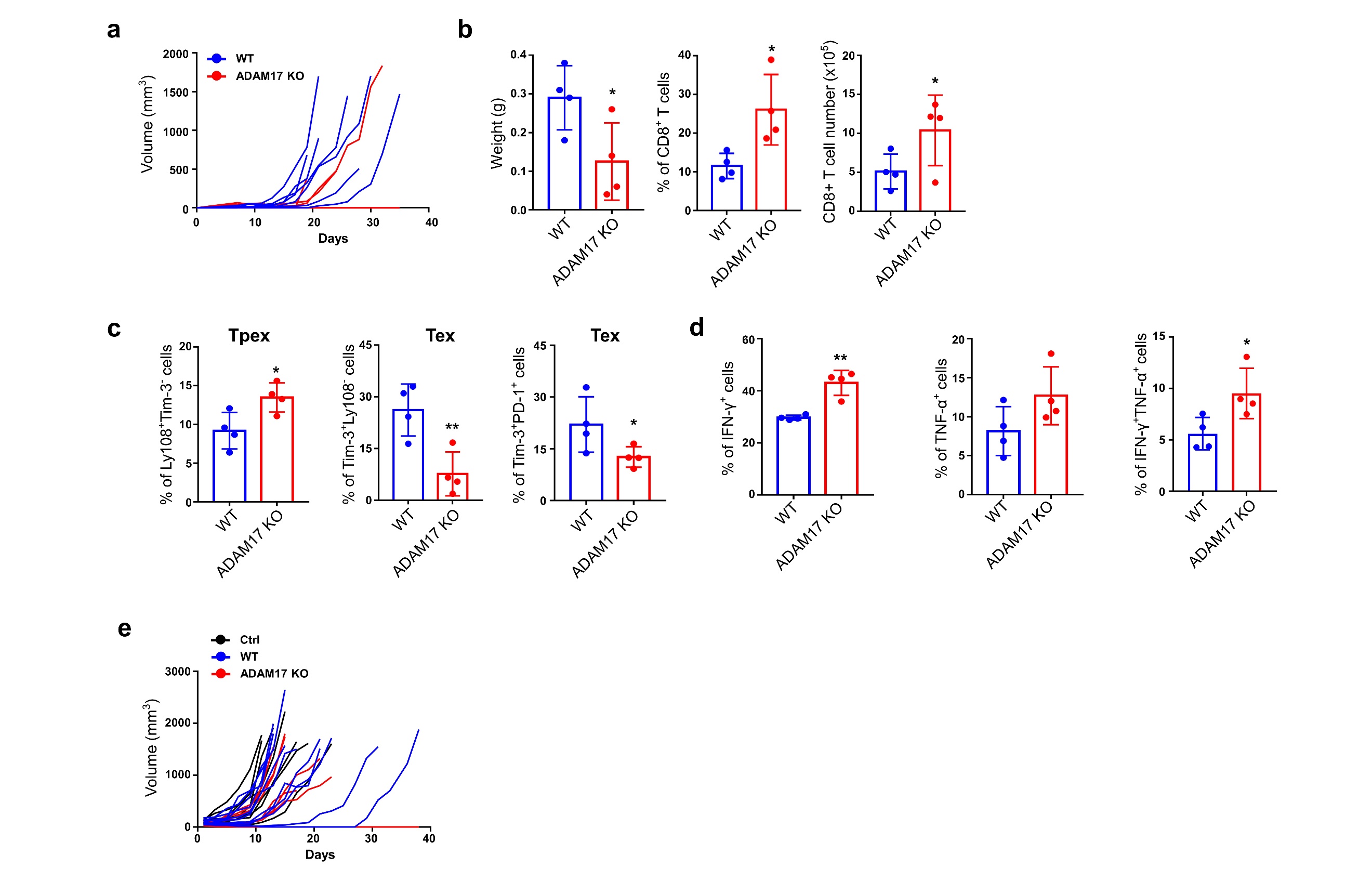


**Figure. S4.**

**ADAM17 deficient CD8^+^ T cells have enhanced anti-tumor immunity.** (**a**) Tumor growth and tumor-infiltrating CD8^+^ T cells were analyzed in the direct tumor model related to **Fig. 3a**. Tumor growth of each individual mouse was monitored over time. (**b**) The tumor weight (left), percentages of CD8^+^ T cells in tumor-infiltrating immune cells (middle) and the total cell count of CD8^+^ T cells (right) were shown in WT and ADAM17 KO mice (n=4). (**c**) The frequencies of Ly108^+^Tim-3^-^, Tim-3^+^Ly108^-^ and Tim-3^+^PD-1^+^ cells in WT and ADAM17 KO CD8^+^ T cells. (**d**) The frequencies of IFN-γ^+^, TNF-α^+^ and IFN-γ^+^TNF-α^+^ cells in WT and ADAM17 KO CD8^+^ T cells. (**e**) Tumor growth of each individual mouse was monitored over time in the ACT tumor model related to **Fig. 3d**. Data are shown as the mean ± SD. Statistical testing is depicted as two-sided, unpaired t-tests; *P ≤ 0.05, **P ≤ 0.01.


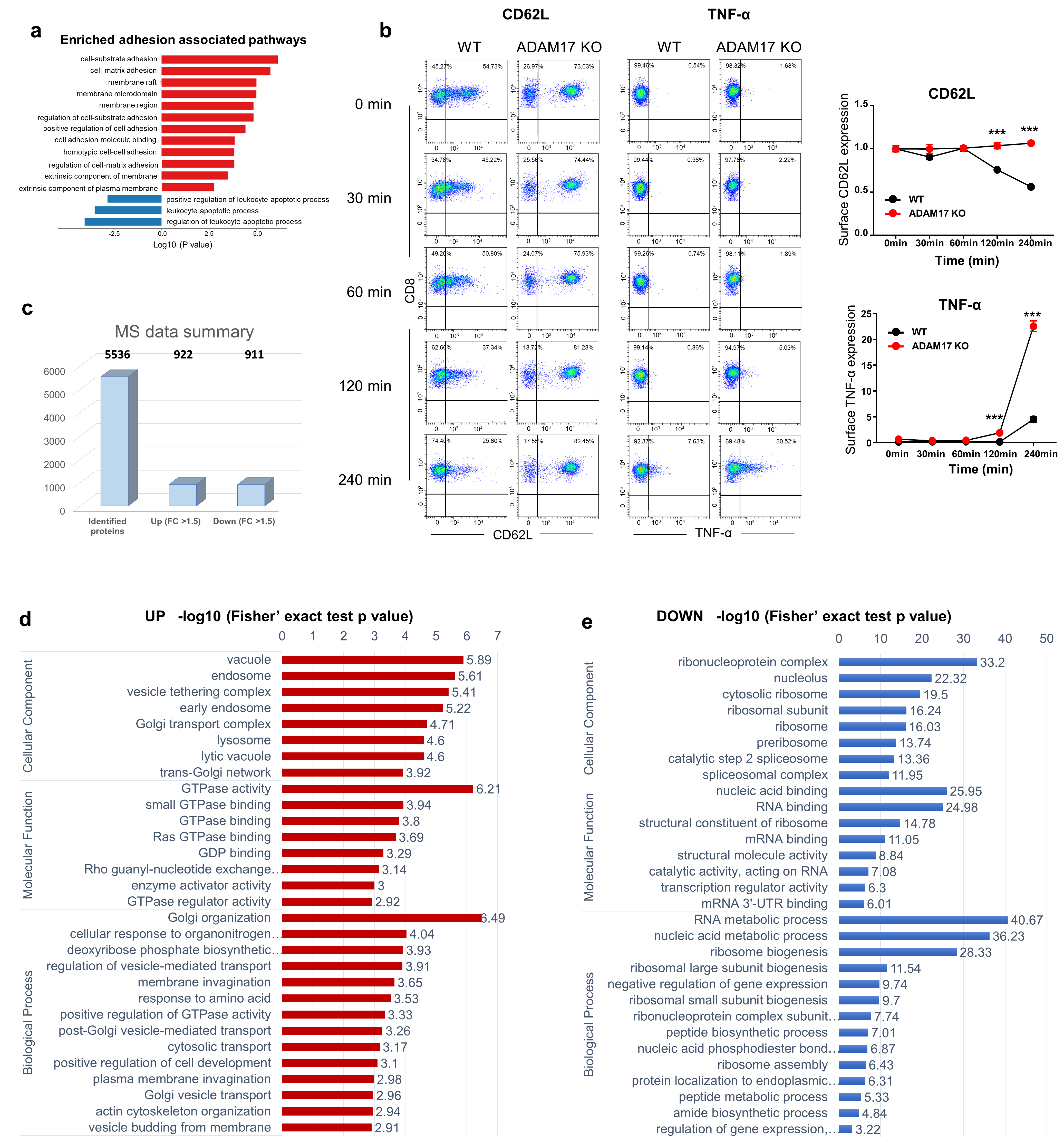


**Figure. S5.**

**ADAM17 deficiency alters the activity of membrane proteins on CD8^+^ T cells.** (**a**) GO analysis of RNA-seq data depicting the genes enriched in ADAM17 KO CD8^+^ T cells were associated membrane activity. (**b**) The representative FACS plots and time kinetics of surface CD62L and TNF-α expression in OT-1^+^ WT and ADAM17 KO CD8^+^ T cells stimulated *in vitro* with OVA peptide for indicated time. (**c**) In the co-transfer model, WT and ADAM17 KO CD8^+^ T cells from recipient mice 7 days after *LM*-OVA infection were sorted and extracted membrane proteins for proteomic analysis (n=3). The number of total identified proteins, upregulated and downregulated (Fold change>1.5) proteins in ADAM17 KO group was shown. GO annotation of cellular component, molecular function and biological process using the upregulated (**d**) and downregulated (**e**) proteins in ADAM17 KO group. Data are shown as the mean ± SD. Statistical testing is depicted as two-sided, unpaired t-tests; ***P ≤ 0.001.


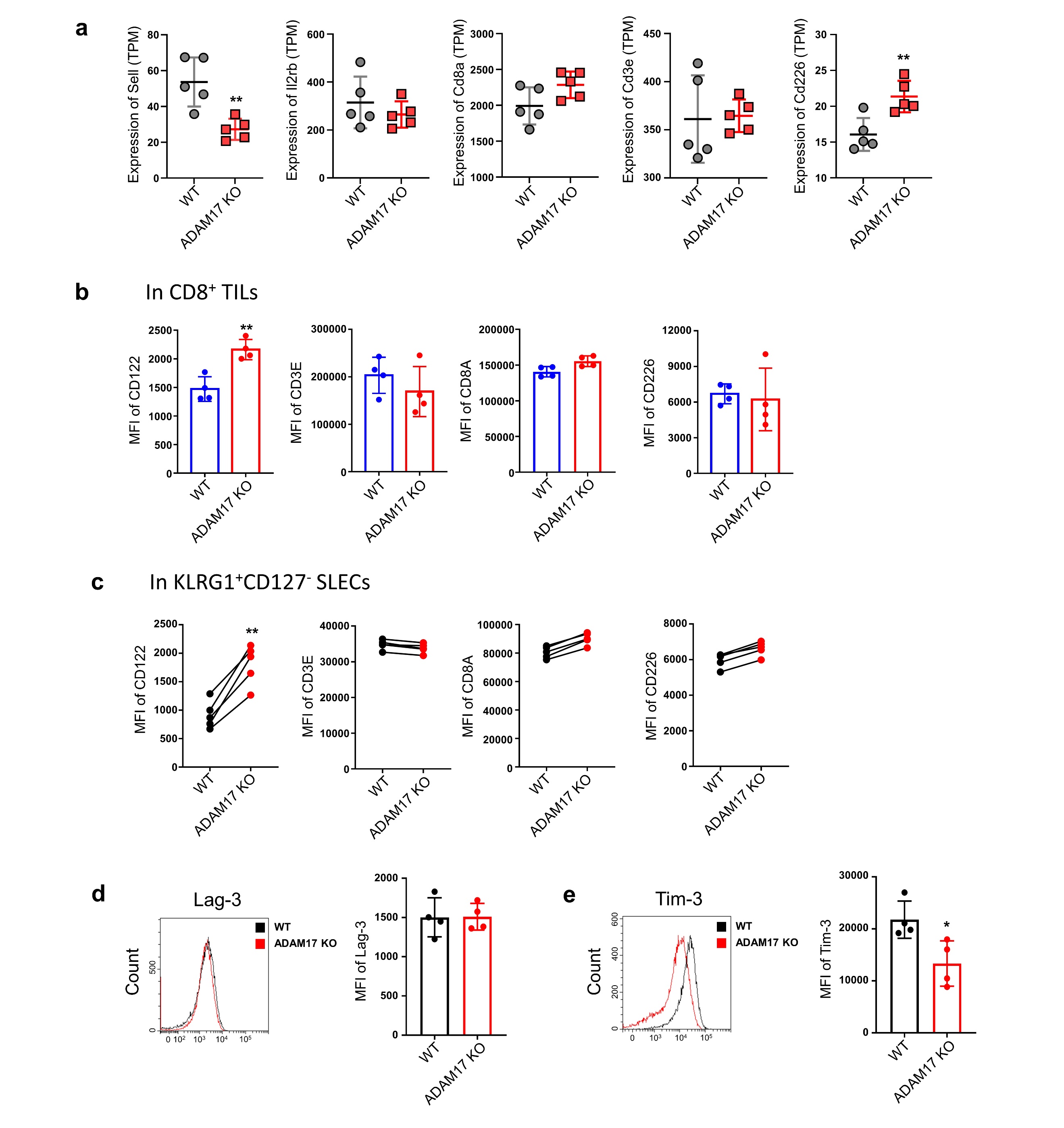


**Figure. S6.**

**The expression of ADAM17 candidate molecules.** (**a**) The mRNA levels of *Sell, Il2rb, Cd8a, Cd3e* and *Cd226* WT and ADAM17 KO CD8^+^ T cells from RNA-seq data. (**b**) The MFI levels of CD122, CD3E, CD8A and CD226 in WT and ADAM17 KO tumor-infiltrating CD8^+^ T cells in the direct tumor model. (**c**) The MFI levels of CD122, CD3E, CD8A and CD226 in WT and ADAM17 KO KLRG1^+^CD127^-^ SLECs from the *in vivo* acute infection model. (**d**-**e**) Representative FACS plots and the MFI levels of Lag-3 (**d**) and Tim-3 (**e**) in WT and ADAM17 KO tumor-infiltrating CD8^+^ T cells in the adoptive cell transfer tumor model. Data are shown as the mean ± SD. Statistical testing is depicted as two-sided, unpaired t-tests; **P ≤ 0.01.

**
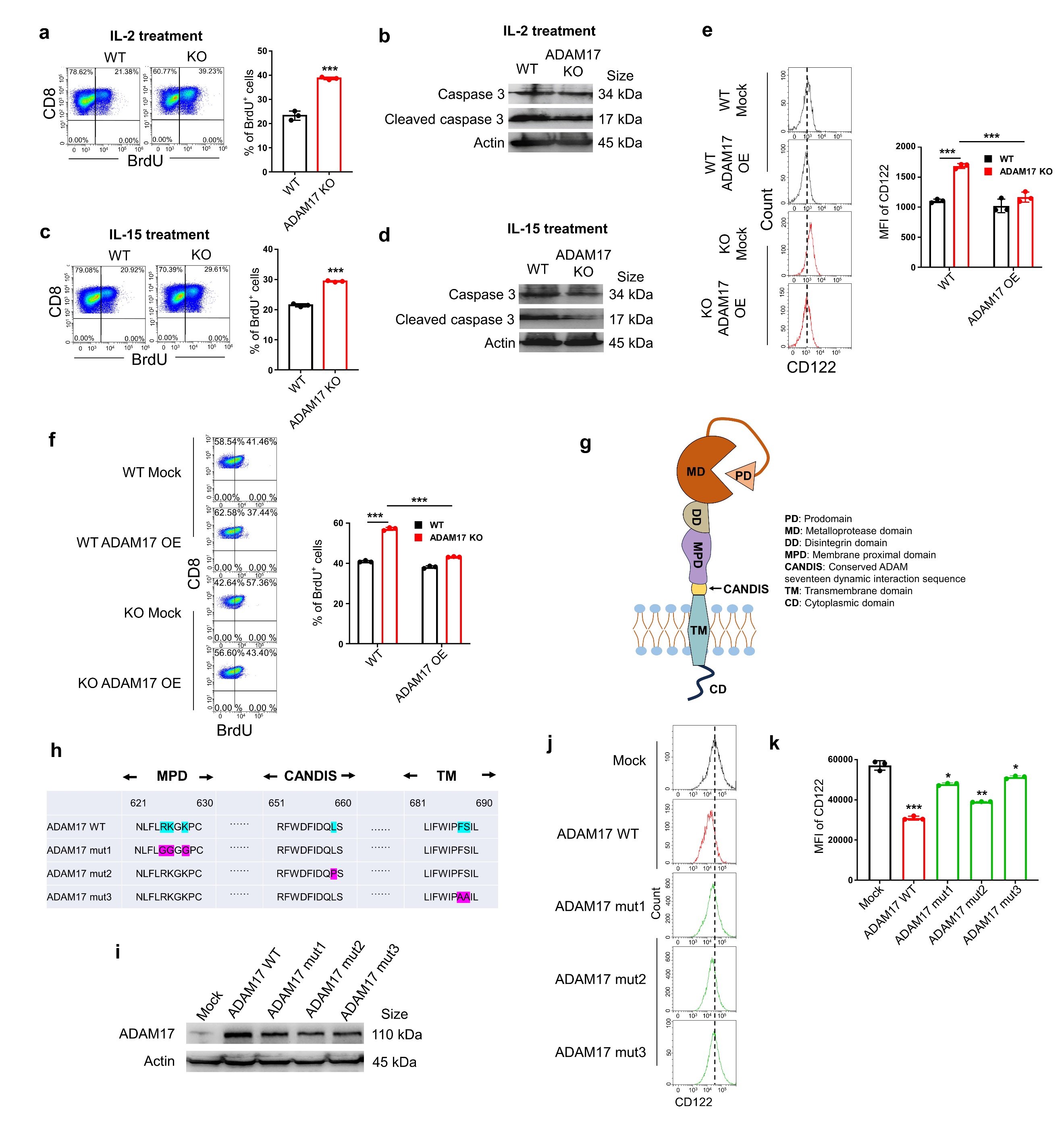
**

**Figure. S7.**

**ADAM17 mediates the ectodomain shedding of CD122 in CD8^+^ T cells.** WT and ADAM17 KO CD8^+^ T cells were stimulated with anti-CD3/CD28 antibodies for 2 days and switched into a culture containing anti-CD3 antibody and cytokines IL-2 or IL-15. Representative FACS plots and the frequencies of BrdU expression in WT and ADAM17 KO CD8^+^ T cells in the presence of IL-2 (**a**) or IL-15 (**c**). The protein levels of total caspase 3 and cleaved caspase 3 were measured by Western blot in WT and ADAM17 KO CD8^+^ T cells in *in vitro* stimulation in the presence of IL-2 (**b**) or IL-15 (**d**). (**e**) WT and ADAM17 KO CD8^+^ T cells were stimulated with anti-CD3/CD28 antibodies for 16hr before transfection with either mock or *Adam17*-overexpressing (OE) retrovirus for 2 days. Representative FACS plots and MFI levels of CD122 in each group were shown. (**f**) WT and ADAM17 KO CD8^+^ T cells were stimulated with anti-CD3/CD28 antibodies for 16hr before transfection with either mock or *Adam17*-overexpressing (OE) retrovirus for 2 days, and switched into culture containing anti-CD3 antibody and cytokines IL-2 for another 2 days. Representative FACS plots and the frequencies of BrdU expression in WT and ADAM17 KO CD8^+^ T cells. (**g**) Schematic representation of the structural and functional domains of ADAM17. (**h**) Schematic representation of WT and ADAM17 variants showing the mutations in MPD (mutant 1: R625G/K626G/K628G), CANDIS (mutant 2: L659P) and TM (mutant 3: F687A/S688A) domains. (**i**) The protein expression of ADAM17 in HEK293 cells after 48hr transient transfection with vectors encoding WT or mutated ADAM17 by Western blot. (**j**-**k**) Representative FACS plots (**j**) and MFI levels (**k**) of CD122 in HEK293 cells after 48hr transient transfection with vectors encoding CD122 together with either WT or mutated ADAM17. Data are shown as the mean ± SD. Statistical testing is depicted as two-sided, unpaired t-tests; *P ≤ 0.05, **P ≤ 0.01, ***P ≤ 0.001.


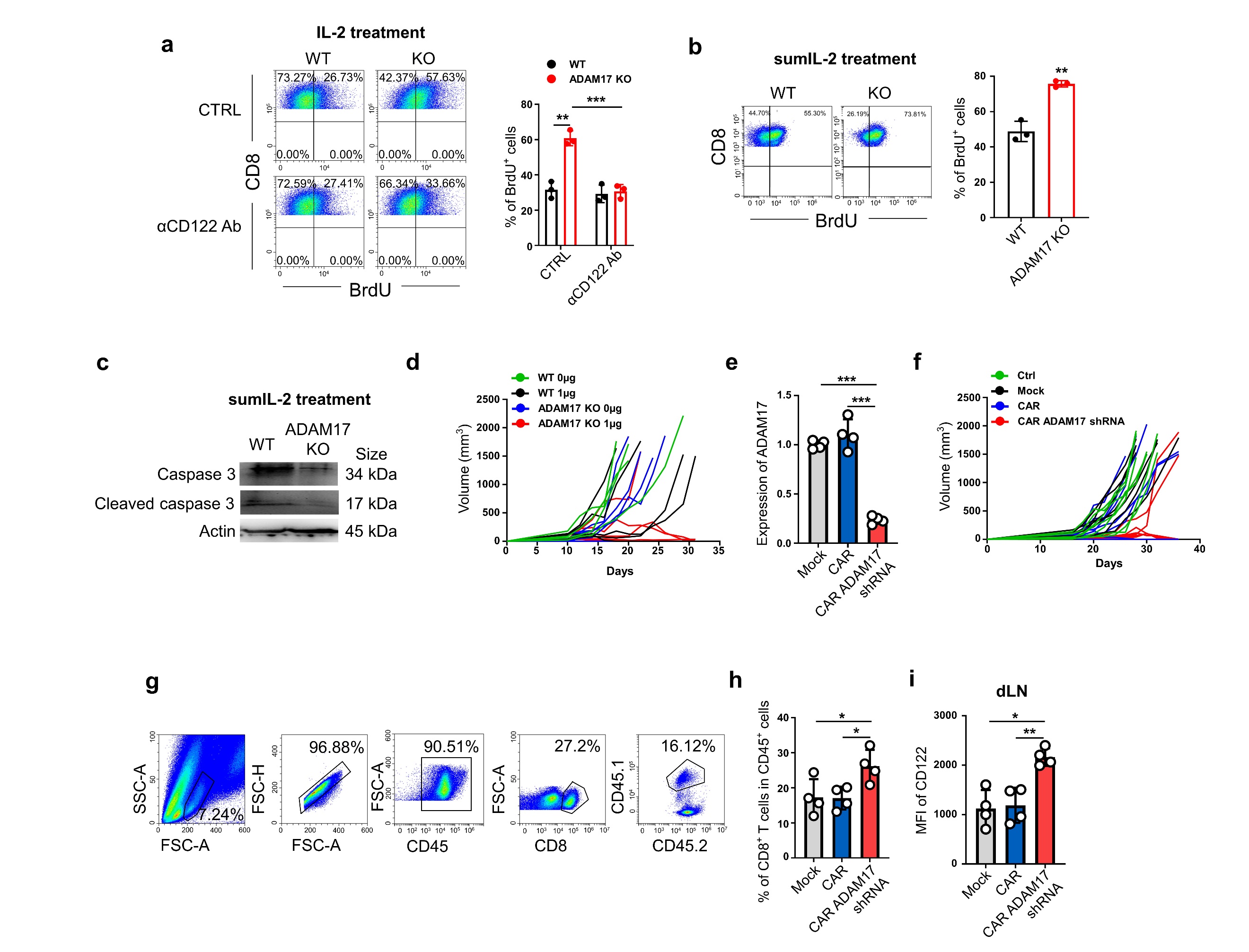


**Figure. S8.**

**ADAM17 depletion promotes CD8^+^ T cell function through enhancing CD122 signaling.** (**a**-**c**) WT and ADAM17 KO CD8^+^ T cells were stimulated with anti-CD3/CD28 antibodies for 2 days and switched into the culture containing anti-CD3 antibody and cytokines IL-2 or sumIL-2 with or without the presence of anti-CD122 blocking antibodies. (**a**) Representative FACS plots and the frequencies of BrdU expression were shown in WT and ADAM17 KO CD8^+^ T cells. (**b**) Representative FACS plots and the frequencies of BrdU expression in WT and ADAM17 KO CD8^+^ T cells in the presence of sumIL-2. (**c**) The protein levels of total caspase 3 and cleaved caspase 3 were measured by Western blot in WT and ADAM17 KO CD8^+^ T cells in the presence of sumIL-2. (**d**) Tumor growth of each individual mouse was monitored over time in the tumor model related to **Fig. 5n**. (**e**) The mRNA levels of ADAM17 in different groups of CAR-T cells were measured by qPCR (n=4). (**f**) Tumor growth of each individual mouse was monitored over time in the CAR-T tumor model related to **Fig. 6c**. (**g**) The gating strategy describing the adoptively transferred CAR-T cells (CD45.1^+^CD45.2^+^) isolated from CD8^+^ TILs for phenotypic assessment. (**h**) The percentage of CD8^+^ T cells among CD45^+^ TILs in different groups of CAR-T cells. (**i**) The MFI level of CD122 expression in CAR-T cells isolated from the draining LN. Data are shown as the mean ± SD. Statistical testing is depicted as two-sided, unpaired t-tests; *P ≤ 0.05, **P ≤ 0.01, ***P ≤ 0.001.
