# Supplementary material for: Targeting a disintegrin and metalloprotease (ADAM) 17-CD122 axis enhances CD8+ T cell effector differentiation and anti-tumor immunity
Source: Signal Transduct Target Ther. 2024 Jun 26;9:152. doi: 10.1038/s41392-024-01873-6 (PMC11199508; doi:10.1038/s41392-024-01873-6)

Fig. 2e   Fig. 2f

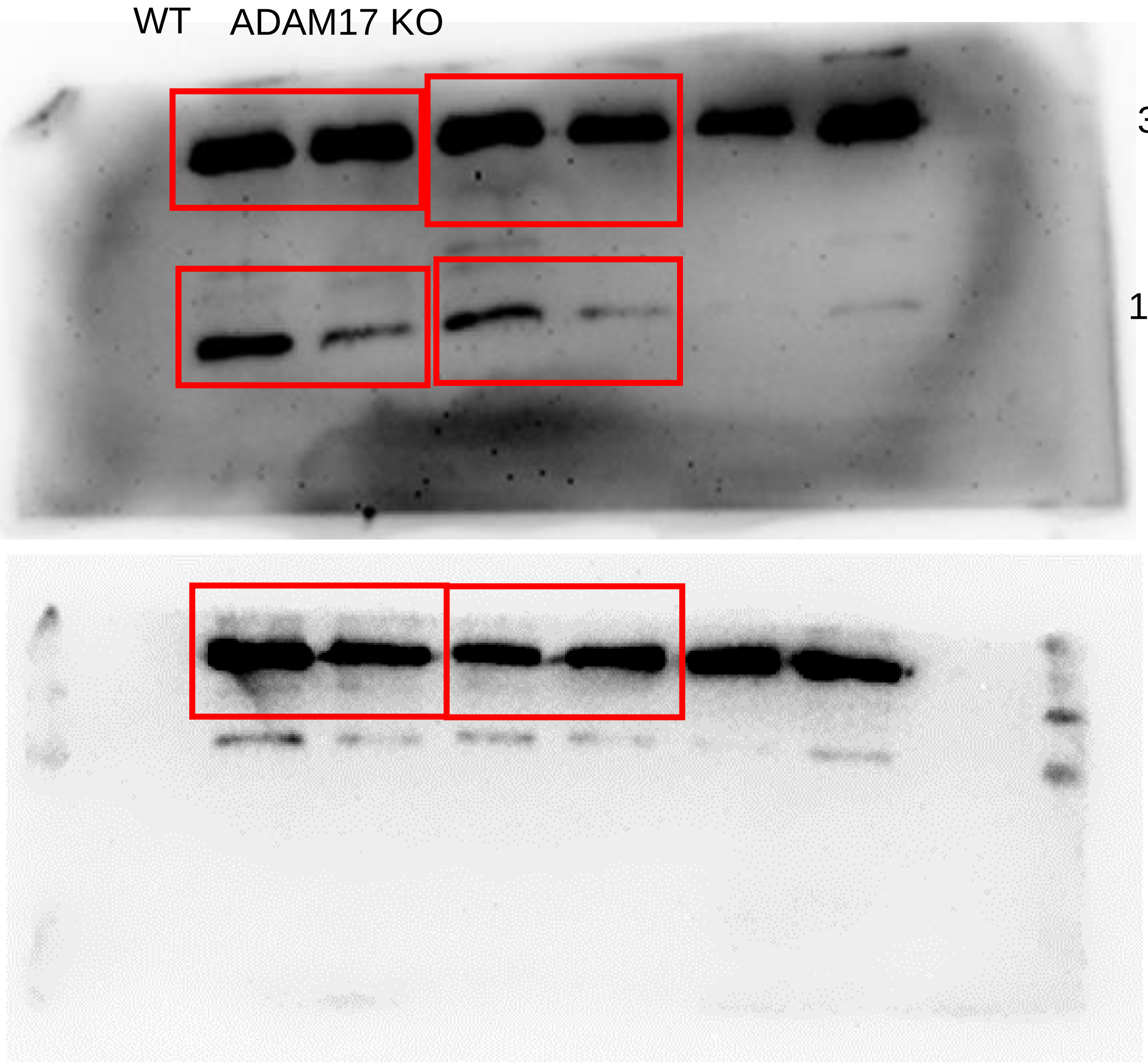

Supplementary Fig. 1e

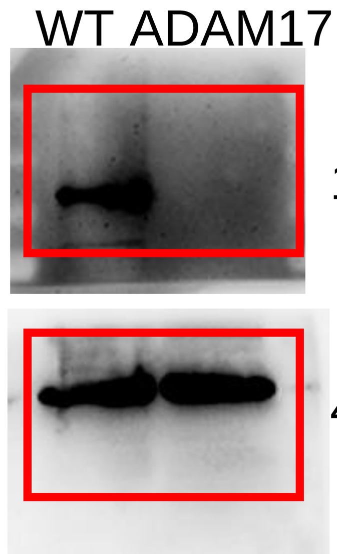

Supplementary Fig. 3c

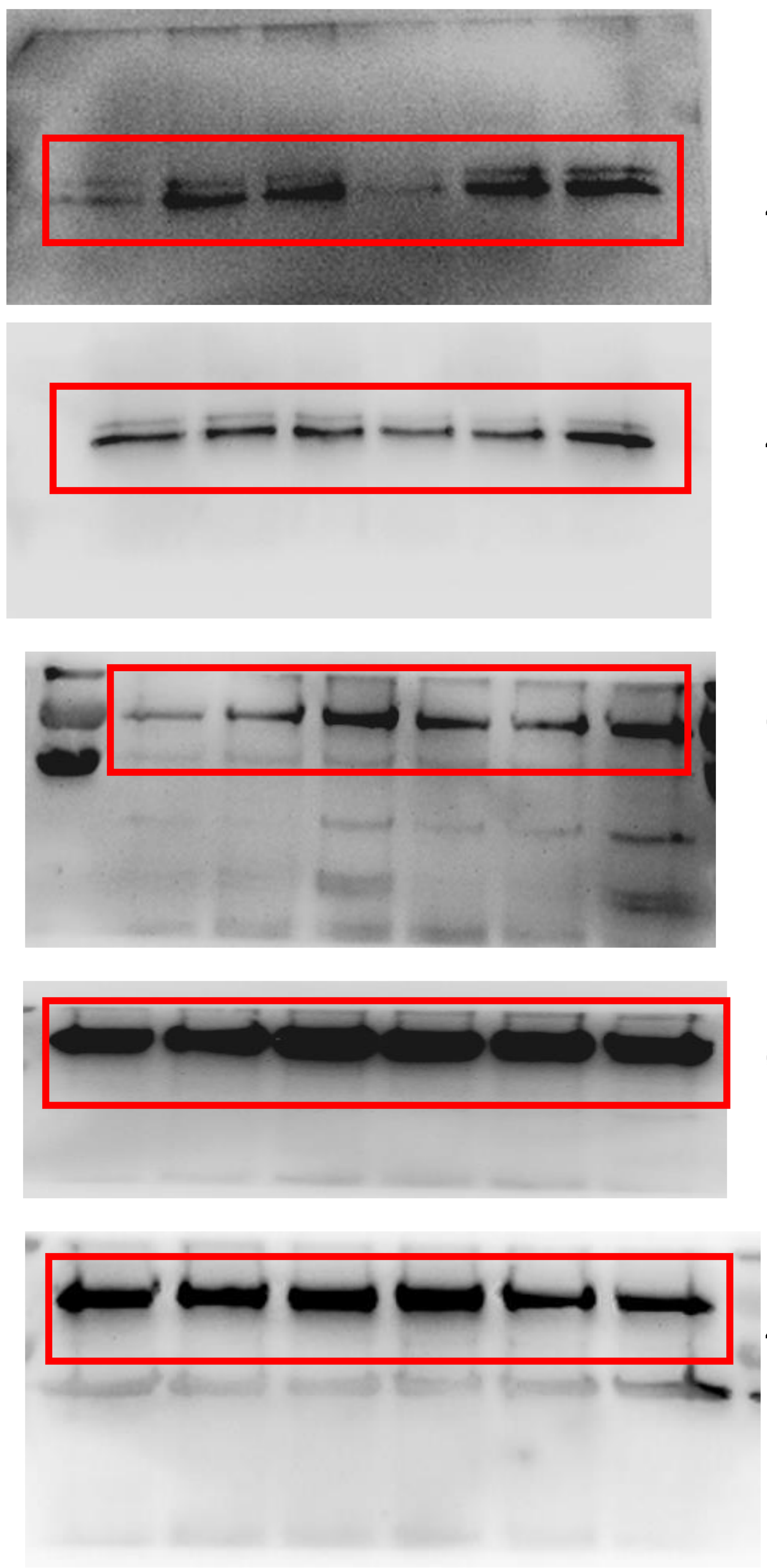

Supplementary Fig. 7b and d

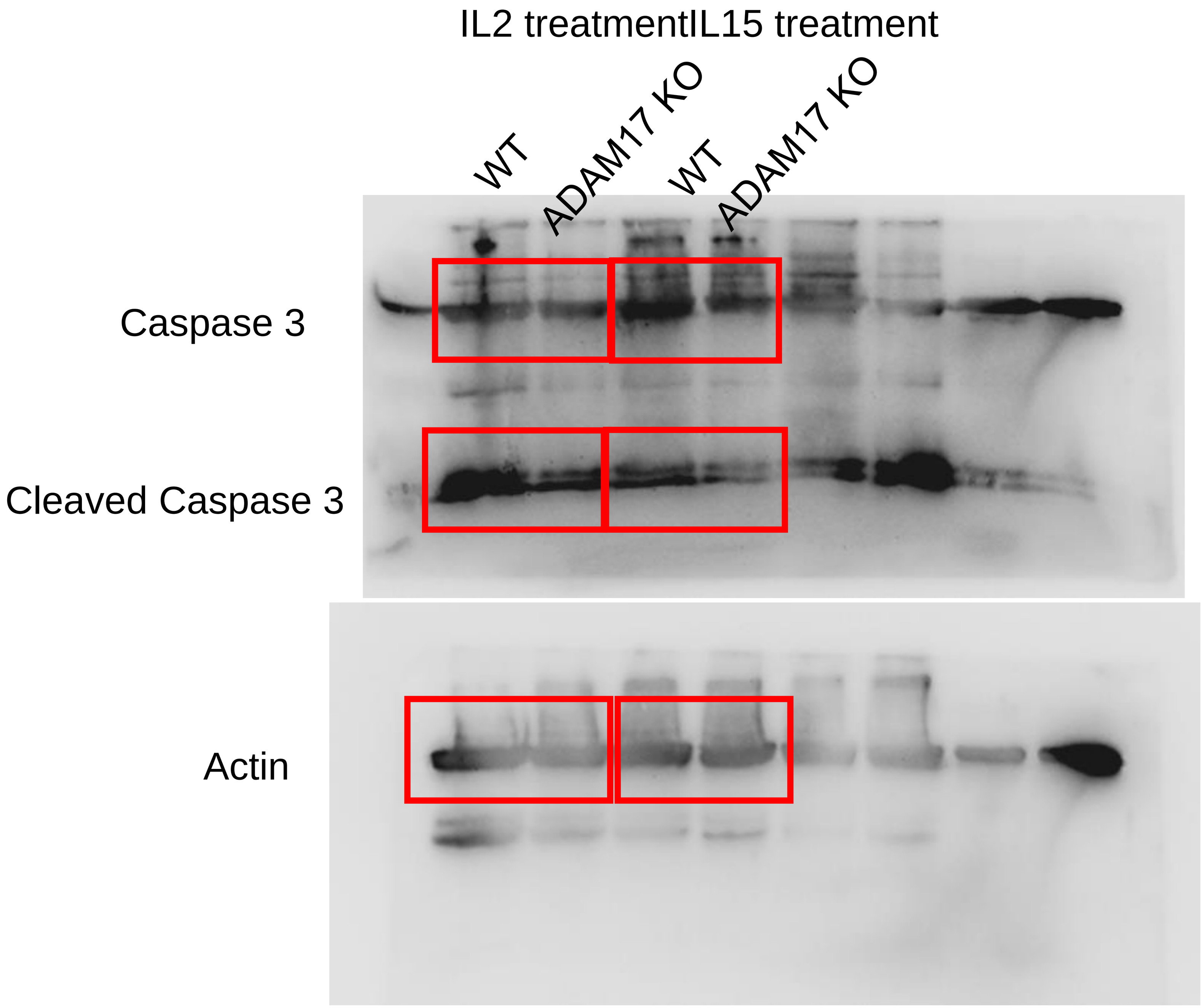

Supplementary Fig. 7i

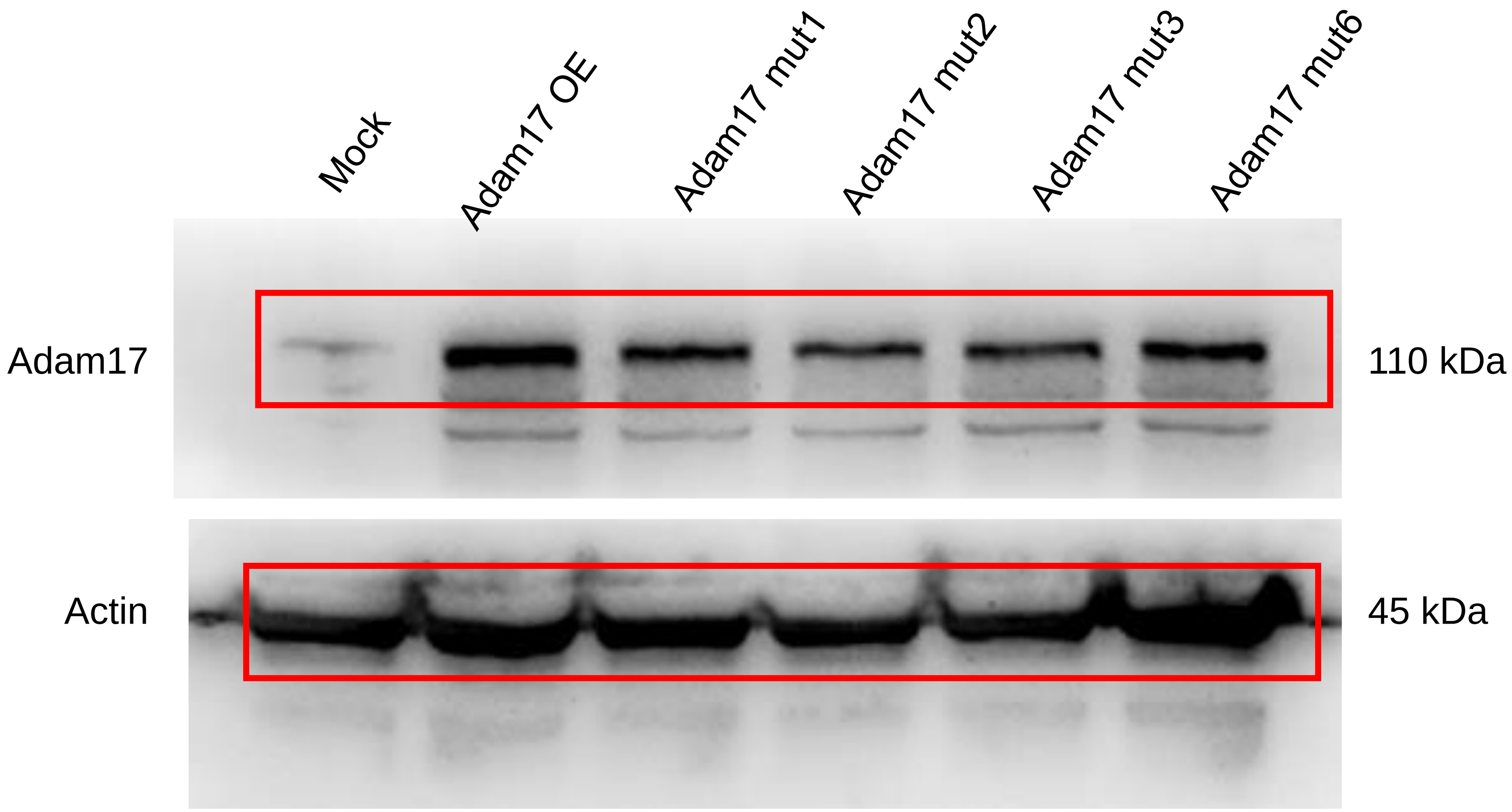

Supplementary Fig. 8c

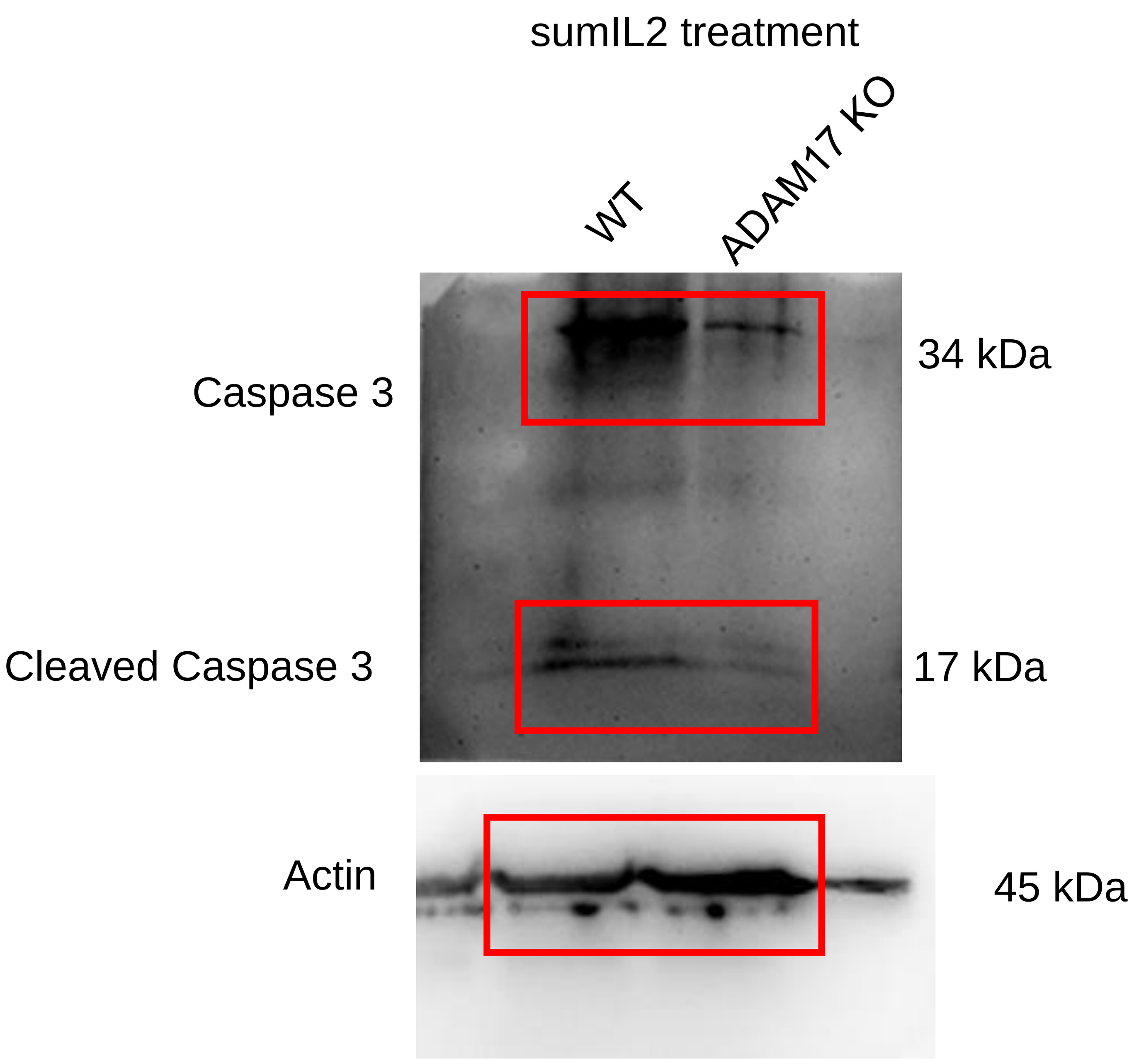

Supplement: Supplementary file 2 — Original western blot figures [file 41392_2024_1873_MOESM2_ESM.pdf]
